# TRPA1 and TRPV1 Receptors Protect the Gastric Mucosa from Ethanol-Induced Injury: Evidence from Knockout Mice

**DOI:** 10.3390/cells15100863

**Published:** 2026-05-09

**Authors:** Michal Zalecki, József Kun, Judyta Juranek, Hanna Antushevich, Zsuzsanna Helyes, Erika Pintér

**Affiliations:** 1 Department of Animal Anatomy, Faculty of Veterinary Medicine, University of Warmia and Mazury, Oczapowskiego 13 Street, 10-719 Olsztyn, Poland; 2 Department of Pharmacology and Pharmacotherapy, Medical School, University of Pécs, 12 Szigeti Street, 7624 Pécs, Hungary; 3 Omics Centre, Szentágothai Research Centre, University of Pécs, 20 Ifjúság Street, 7624 Pécs, Hungary; 4 Department of Human Physiology and Pathophysiology, School of Medicine, Collegium Medicum, University of Warmia and Mazury, Warszawska 30 Street, 10-082 Olsztyn, Poland; 5 Department of Genetic Engineering, The Kielanowski Institute of Animal Physiology and Nutrition, Polish Academy of Sciences, Instytucka 3 Street, 05-110 Jabłonna, Poland; 6 National Laboratory for Drug Research and Development, 1 Kéthly Anna Square, 1077 Budapest, Hungary; 7 HUN-REN-PTE Chronic Pain Research Group, 12 Szigeti Street, 7624 Pécs, Hungary

**Keywords:** TRP receptors, ethanol, gastric injury, stomach, mucosa, KO animals

## Abstract

**Highlights:**

**What are the main findings?**

- TRPA1 and TRPV1 exert protective effects against ethanol-induced gastric injury.
- Gastroprotection is gradational and strongest when both receptors coexist.

**What are the implications of the main findings?**

- TRPA1 and TRPV1 may represent potential targets for protecting the gastric mucosa from injury.
- Adaptive regulation of these receptors suggests a role in gastric tissue responses to harmful stimuli.

**Abstract:**

Ethanol disrupts gastric mucosal integrity and induces inflammation. Transient receptor potential ankyrin 1 (TRPA1) and transient receptor potential vanilloid 1 (TRPV1) are receptors expressed in the gastrointestinal tract, but their role in gastric protection against ethanol-induced injury remains unclear. This study investigated the contribution of TRPA1 and TRPV1 to gastric responses following ethanol exposure. Wild-type mice and mice lacking *TRPA1*, *TRPV1*, or both receptors were subjected to intragastric ethanol administration. Gastric injury was evaluated by macroscopic and histologic analysis. Messenger RNA expression of selected proinflammatory cytokines and receptor transcripts was quantified using quantitative polymerase chain reaction. Receptor localization was examined by double immunofluorescence and confocal microscopy. Ethanol administration induced pronounced gastric mucosal injury in receptor-deficient mice, whereas only minimal changes were observed in wild-type animals. The protective effect was gradational, with weaker protection associated with TRPV1, greater protection with TRPA1, and the strongest protection when both receptors were present, suggesting a combined contribution of both receptors. In wild-type animals, ethanol exposure induced time-dependent changes in receptor expression, suggesting adaptive regulation. Immunofluorescence revealed localization of both receptors in neuronal and non-neuronal structures of the gastric wall. These findings demonstrate the distinct roles of TRPA1 and TRPV1 in protecting the gastric mucosa against ethanol-induced injury.

## 1. Introduction

As a reservoir for ingested food, the stomach is directly exposed to potentially harmful substances. Due to its role in mechanical and chemical digestion in a strongly acidic environment, these factors may disrupt mucosal homeostasis and lead to gastric lesions. One of these substances is ethanol, a common component of beverages consumed by humans.

Natural medicines and spices have long been believed in various cultures to play a gastroprotective role. Interestingly, according to some authors, certain species provide even stronger protection against ethanol-induced gastric lesions than that of an antiulcer drug [1,2,3,4,5]. To the group of plants considered gastroactive belong, among others, horseradish [6] and chili pepper [3,7,8] containing allyl isothiocyanate and capsaicin, respectively, as their active compounds. Both components act on members of the transient receptor potential (TRP) family of nonselective cation channels. In detail, allyl isothiocyanate acts on ankyrin (TRPA1), while capsaicin acts on the vanilloid (TRPV1) receptor. Both receptors are additionally activated by a variety of chemical and physical stimuli and play important roles in both physiological and pathophysiological processes [9]. Although most studies agree on the gastroprotective role of capsaicin and TRPV1 activation, there are also reports indicating its pro-inflammatory role in the gastrointestinal mucosa, such as under indomethacin-induced gastric ulcerations [10]. New evidence also highlights natural compounds that modulate TRP receptors and exhibit gastroprotective effects [11,12]. Importantly, results on the TRPA1 mucosal protective function are not consistent, and according to many researchers, its role depends on the main pathomechanism of mucosal inflammation and the part of the gastrointestinal tract affected by the lesion. Thus, TRPA1 activation may frequently act as a proinflammatory factor [13,14,15,16] or as an anti-inflammatory agent [3,17]. Since receptors often co-exist on the same neural cells [18,19], the stimulation of one receptor may be indirectly influenced by the action of another. Therefore, the use of genetically modified animals lacking individual receptors seems to be the best method to accurately verify the role of each receptor in ethanol-induced gastritis.

The regulation of receptor expression is one of the key mechanisms ensuring the appropriate response of tissues to pathological factors. Therefore, understanding receptor changes under pathological conditions may help understand the functional regulation of the pathology. In line with this, dysregulation in TRP receptor function has been found to be associated with a number of disorders [9]. Therefore, the verification of changes in gastric TRPA1 and TRPV1 receptors expression following alcohol administration in wild-type animals, together with their time-dependent changes, could reveal the adaptive changes of gastric tissues to ethanol, ensuring homeostasis. Moreover, evaluating the response of the remaining receptor in knockout animals may help determine whether the organism attempts to activate compensatory mechanisms.

Both receptors are known to be present on extrinsic sensory neurons supplying the gastrointestinal tract and are often co-expressed in the same neurons [19]. According to the widely accepted theory, these neurons primarily function in pain transduction, including sensitivity to noxious cold (TRPA1) and heat (TRPV1) [20,21,22,23]. However, their protective role in the gastrointestinal mucosa is mainly associated with their activation, which triggers the release of neuropeptides (such as CGRP) from the peripheral nerve endings of sensory neurons [24,25]. The co-expression of both receptors in nociceptive sensory neurons supplying the gastric wall suggests that the gastroprotective effect induced by TRPV1 agonists may be interpreted and linked to TRPA1 activation, and vice versa. The use of separate groups of knockout animals for *TRPA1*, *TRPV1* and double *TRPA1/TRPV1* allows for a precise distinction of each receptor’s mode of action, as well as indicates possible interactions and compensatory mechanisms between both receptors. Since the expression of TRPA1 and TRPV1 has occasionally been reported on non-neuronal structures of the gastrointestinal tract [13,26,27], non-neuronal mechanisms of gastroprotective function should also be considered. The application of immunofluorescent staining combined with confocal microscopy in the stomach of wild-type animals appears to be a method of choice, despite its limitations, to understand the localization of both receptors in the gastric wall.

Although TRPA1 and TRPV1 are widely associated with sensory neuronal signaling in the gastrointestinal tract, it remains unclear whether their protective effects in the stomach are mediated exclusively by neuronal pathways or also involve non-neuronal gastric cell populations.

Therefore, the aim of the present study was to evaluate the role of TRPA1 and TRPV1 in the gastric mucosal response to ethanol, with particular emphasis on distinguishing their individual contributions and potential interactions. In addition, the study aimed to assess whether the protective effects of these receptors differ in magnitude and whether their expression undergoes adaptive changes in response to ethanol exposure.

To address these questions, we applied an integrated approach combining a comparative analysis of receptor-dependent effects in single and double knockout models with the evaluation of gastric mucosal damage, inflammatory responses, and receptor expression dynamics, together with their anatomical localization within both neuronal and non-neuronal structures of the gastric wall.

This approach allows for a comprehensive assessment of receptor-specific effects and supports the concept that TRPA1 and TRPV1 may contribute to gastric mucosal protection through both neuronal and non-neuronal mechanisms.

## 2. Materials and Methods

### 2.1. Animals

Experiments were carried out using *TRPV1* receptor gene knockout (KO), *TRPA1* KO and *TRPA1V1* double knockout (dKO) mice (6 months old, 20–25 g) and their wild-type counterparts (WT). All details on the animals’ strains, their origin, crossbreeding, and testing were precisely described in previous articles [17,28,29]. All animals were bred and kept in standard polycarbonate cages (365 mm × 207 mm × 104 mm) in groups of four to six mice per cage (temp. 24 °C; 12-h light-dark cycle; provided with standard rodent chow and water ad libitum) in the conventional animal house of the Department of Pharmacology and Pharmacotherapy of the University of Pécs.

All procedures at the University of Pécs were carried out according to the 1998/XXVIII Act of the Hungarian Parliament on Animal Protection and Consideration Decree of Scientific Procedures of Animal Experiments (243/1988). All experiments were approved by the Ethics Committee on Animal Research of the University of Pécs, according to the Ethical Codex of Animal Experiments. The license number is BA02/2000-25/2021.

### 2.2. Experimental Design

The experiment was performed on *TRPV1* KO (*n* = 12), *TRPA1* KO (*n* = 12), *TRPA1V1* dKO (*n* = 12), and WT (*n* = 24) mice, each randomly divided in half into the experimental and control groups. Additionally, the wild-type animal groups were further subdivided into two subcategories, WT2 and WT5, tested accordingly for 2 and 5 consecutive days, respectively. The remaining knockout groups were subjected to the procedure only for 2 consecutive days.

In experimental animals [*TRPV1* KO (*n* = 6), *TRPA1* KO (*n* = 6), *TRPA1V1* dKO (*n* = 6), WT2 (*n* = 6)], 150 µL of 50% EtOH was administered by oral gavage for 2 consecutive days following a modified protocol described previously [30]. Distilled water was given to animals from control groups (*n* = 6 mice in each group) in the same manner. In the WT5 subcategory, the animals EtOH (experimental mice) and distilled water (control mice) in the same manner for 5 consecutive days. The gastric gavage procedure was performed at the scheduled time (starting at 12:00). Four hours earlier, the animals were deprived of access to feed and water (at 8:00) and weighed. Half an hour after the treatments, the animals were given unlimited access to food and water again.

Twenty-four hours after the last gavage procedure, the animals were weighed and euthanized under deep ketamine–xylazine anesthesia (120 mg/kg ketamine ip.; Calypsol, Gedeon Richter Plc., Budapest, Hungary; 6 mg/kg xylazine ip.; Sedaxylan, Eurovet Animal Health B.V., Bladel, The Netherlands). Next, the abdomen was opened, and the stomach was removed, cut along the greater curvature and deprived of food debris. Then, the stomach was washed 3 times in phosphate buffer solution, gently drained with tissue paper and placed on the smooth surface of a glass plate with the mucous membrane facing upwards. The prepared stomach tissue was photographed with a digital camera with certain constant parameters. Afterwards, the stomach was completely cut into two corresponding sample parts (along the lesser curvature), one of which was placed in a buffered 4% paraformaldehyde solution (for hematoxylin and eosin staining and immunofluorescence studies), while the other part was placed in RNA later (Thermo Fisher Scientific, Waltham, MA, USA) for 24 h at 4 °C and then frozen at −80 °C (for Q-PCR studies).

Samples immersed in PFA solution were incubated for 1 h, washed for 20 min in phosphate buffer, and finally transferred to an 18% sucrose solution for the time needed for them to be completely saturated with sucrose (2 days). All these steps were performed at 4 °C. Next, these stomach tissues were cryo-sectioned using a Leica CM1520 (Leica Biosystems, Nußloch, Germany) cryostat into 10 µm thick longitudinal sections, mounted on chrome alum-gelatin coated slides, air-dried and frozen at −22 °C for future analysis.

### 2.3. Macroscopic Evaluation of EtOH-Induced Gastric Lesions

The extent and severity of the macroscopic lesions were evaluated by a quantitative analysis using ImageJ software version 1.54 (National Institutes of Health, Bethesda, MD, USA) on photographs of the stomach glandular mucosa. The percentage of mucosal area covered by lesions and the difference in the gray value between lesioned and normal mucosa were calculated for each stomach of the EtOH-administrated animals in each studied group. Using the ROI and XOR functions, the proportion of the area covered with lesions in relation to normal mucosa was established and presented as a percentage for each photograph studied. By comparing the gray value estimated by the ImageJ software analysis for the lesioned mucosa in relation to unchanged mucosa, the percentage of gray value difference was estimated, which corresponded to the intensity of pathological changes. To obtain the most valuable results, the analysis was performed on 3 different pictures for each studied stomach and the results were averaged for each stomach/each animal. Results obtained from all the stomachs in each alcohol-administrated group were averaged and presented as a percentage ± SEM. Finally, the results obtained in each group were statistically analyzed to compare the intergroup differences.

### 2.4. Hematoxylin & Eosin Staining and Microscopic Evaluation

The optimal duration of H&E staining intensity was obtained in preliminary tests by controlling the tissues through visual examination with a light microscope. For the final assessment, corresponding air-dried tissue slides from three mice of each ethanol-treated animal group and one mouse from the water-treated group (control animal) were stained with eosin–hematoxylin solution according to Ehrlich (Fluka BioChemika, Fluka Chemie AG, Buchs, Switzerland; cat. no. 09372) for 15 min (and then rinsed under running water for 15 min). Slides were finally cover-slipped with glycerol and analyzed under a Zeiss optical microscope (Axio Scope A1, Carl Zeiss Microscopy GmbH, Germany) equipped with a Zeiss digital camera (AxioCam HRc, Carl Zeiss Microscopy GmbH, Germany) to evaluate microscopic pathological changes in the stomach wall. Longitudinal sectioning enabled the analysis of all stomach regions (cardia, body, pylorus) within a single tissue slice, allowing for an objective assessment of changes in different parts of the stomach. The researcher was blinded to the animal type from which the tissue sample on the slide originated. Observed pathological changes were described, and selected areas were photographed as part of the photographic documentation.

### 2.5. Immunofluorescence and Microscopic Evaluation

Experience with receptor immunostaining and the associated challenges led us to conduct preliminary analyses before the final design of the immunofluorescent part of the studies. All antibodies used in the study were selected on the basis of experience from previously conducted immunocytochemical studies with these antigens. Moreover, due to the high complexity of receptor staining, the TRPA1 and TRPV1 antibodies were validated bioinformatically for the compatibility of the antigens used in the antibody production with the amino acid sequences of mouse tissues to confirm their best matching. Furthermore, a series of control stainings were conducted to distinguish the specificity of the stained structures from the non-specific background. In addition to standard antibody controls (replacement and omission), supplementary sets of control staining with mixtures of primary and secondary antibodies were performed on tissues obtained from double knockout animals (*TRPA1V1* dKO) to further verify antibody specificity.

All these controls revealed the potential for a slight nonspecific immunofluorescence signal despite the use of the best available antibodies for this type of study. This limitation excluded the possibility of conducting comparative studies on tissues from different types of knockout animals, and therefore, we decided to perform analyses solely on tissues from wild-type control mice to reliably determine the localization of immunofluorescence for the studied receptors within the stomach.

For the general assessment of TRPA1 and TRPV1 localization within the stomach tissue, the double immunostaining procedure was applied to 6 tissue slides of wild-type water-treated (control) mice. Since both receptors are considered to be primarily expressed in neurons, each set of primary antibody mixtures included the anti-pan neuronal marker (PGP 9.5) antibody (mouse monoclonal anti-PGP 9.5, type IgG1, 7863-1004, dilution 1:500, Bio-Rad, Hercules, CA, USA) as a constant component, and additionally anti-TRPA1 (rabbit polyclonal IgG, ab68848, dilution 1:2000, Abcam, Cambridge, UK) or anti-TRPV1 (rabbit polyclonal IgG, 2233; dilution 1:2000; Tocris Bioscience, Bristol, UK) antibody.

The standard double immunofluorescence procedure [31,32] was applied to the selected tissue slides. Briefly, the slides were defrosted, air-dried, rehydrated in PBS, and blocked with the blocking solution (0.25% Triton X-100, 1% bovine serum albumin and 10% normal goat serum in PBS) for one hour (room temperature—RT). Next, the slides were rinsed in PBS, drained, and covered with one of two previously prepared primary antibody mixtures: mouse anti-PGP 9.5 plus rabbit anti-TRPA1, or mouse anti-PGP 9.5 plus rabbit anti-TRPV1 (24 h, RT, humid chamber). On the next day the slides were thoroughly rinsed in PBS (3 × 15 min), drained by suction pump and covered with a mixture of fluorescent secondary antibodies for 1 h (RT, dark humidified chamber). As secondary antibodies, the mix of fluorescent AlexaFluor 488 (Goat anti-Mouse IgG1 Cross-Adsorbed, A-21121, dilution 1:500, Thermo Fisher Scientific, Waltham, MA, USA) and AlexaFluor 555 (Goat anti-Rabbit IgG (H+L) Cross-Adsorbed Secondary Antibody, A-21428, Thermo Fisher Scientific, Waltham, MA, USA) was used. The application of the IgG1 primary antibody with the anti-IgG1 cross-adsorbed secondary antibody ensured high-quality specific staining on mouse tissues.

Finally, the slides were thoroughly rinsed in PBS (3 × 15 min), drained by suction pump and cover-slipped with glycerol mounting medium.

Immunolabeled sections were analyzed under a confocal microscope (LSM 700, Carl Zeiss Microscopy GmbH, Germany), as previously described [33], by a researcher blinded to the tissue’s animal origin. Longitudinal sectioning enabled the analysis of all stomach regions (cardia, body, pylorus) within a single tissue slice, allowing for an objective assessment of immunolabeled structures throughout the entire stomach wall.

Changing the laser and corresponding detectors in the confocal microscope during tissue observations [34] allowed for the verification of the localization of structures stained by the PGP 9.5 antibody (i.e., neural elements and enteroendocrine system cells) and their correlation with structures stained with antibodies against TRPA1 and TRPV1. The analysis confirmed the presence of TRPV1- and TRPA1-immunoreactive structures in different parts of the stomach of water-treated wild-type animals.

Microphotographic confocal laser documentation, including images of individual scanned channels and their resulting merged images, was prepared to illustrate the observations.

### 2.6. Real-Time PCR

Isolation of total RNA was performed according to the manufacturer’s kit protocols. Stomach tissue samples for q-PCR analysis were homogenized in 1 mL of TRI Reagent (Molecular Research Center, Inc., Cincinnati, OH, USA), followed by the addition of 200 µL of bromo-chloro-propane (BCP, Molecular Research Center, Inc., Cincinnati, OH, USA). RNA purification from the aqueous phase was carried out using the Direct-zol RNA MiniPrep kit (Zymo Research, Irvine, CA, USA) according to the manufacturer’s instructions.

During this process, 400 µL of the aqueous supernatant phase was mixed with 400 µL of absolute ethanol. The mixture was then loaded onto the column, washed multiple times, and finally the RNA was eluted in 50 µL of RNase-free water. The quantity and purity of the extracted RNA were assessed using a Nanodrop ND-1000 Spectrophotometer V3.5 (NanoDrop Technologies, Inc., Wilmington, DE, USA). Next, the isolated RNA was purified from DNA residues with the DNase I RNase-free kit (cat. No. 89836, Thermo Fisher Scientific, Waltham, MA, USA) following the manufacturer’s protocol.

Subsequently, 1 µg of total DNA-free RNA was reverse-transcribed into cDNA using the Maxima First Strand cDNA Synthesis Kit (cat. no. K1642, Thermo Fisher Scientific, Waltham, MA, USA) according to the manufacturer’s instructions. The obtained cDNA samples were amplified using the MX3000P qPCR system (Agilent Technologies, Santa Clara, CA, USA), master mix (cat. no. BIO-94005 SensiFAST™ SYBR^®^ Lo-ROX Kit, Meridian Biosience, Cincinnati, OH, USA) and appropriate primers (Table 1).

Initial validation of samples obtained from experimental and control animals confirmed that glyceraldehyde 3-phosphate dehydrogenase (Gapdh) was an appropriate and reliable housekeeping gene for the study, as its expression remained stable across all animals. PCR cycle parameters were set as instructed by the manufacturer of the master mix and matched to the primers used. The reaction protocol was as follows: an initial denaturation at 95 °C for 2 min, followed by 40 cycles of 95 °C for 5 s (denaturation), 62 °C for 10 s (annealing), and 72 °C for 10 s (elongation).

For melting curve analysis, the dissociation characteristics of double-stranded DNA during heating were examined under the following conditions: 1 min at 95 °C, 30 s at 55 °C and 30 s at 95 °C. The efficiency values of the primers were determined to be between 95 and 105%. cDNA samples were measured in duplicate, and the geometric means of duplicate Ct values were calculated to minimize technical variability and account for biological variability. Relative gene expression was calculated using the ΔΔCt method. During the analysis, the log fold change (logFC), SEM, normalized means, and linearized means were calculated, enabling the presentation of data as fold changes in gene expression between ethanol- and water-administered animals in each studied group. The entire procedure, including RNA isolation, cDNA synthesis, and qPCR analysis, was performed according to previously described protocols [17].

### 2.7. Statistical Analysis

Results from each group were expressed as means ± SEM. Results from the control (H_2_O-treated) and experimental (EtOH-treated) animals in each tested group were statistically analyzed using GraphPad Prism software version 6.05 (GraphPad Software Inc., San Diego, CA, USA).

For qPCR data, normality was assessed using the D’Agostino–Pearson test. Depending on data distribution, either an unpaired t-test was applied for normally distributed data or a Mann–Whitney *U* test was used for non-normally distributed data.

For ImageJ-derived data, normality (Shapiro–Wilk test) and homogeneity of variances (Levene’s test) were evaluated prior to selecting the appropriate statistical approach. Parametric analysis was performed using one-way ANOVA with Tukey’s HSD when assumptions were met. Otherwise, non-parametric analysis was performed using the Kruskal-Wallis test followed by pairwise Mann–Whitney *U* tests with Holm correction.

For predefined ordered hypotheses (WT2 → WT5 → TRPV1 KO → TRPA1 KO → TRPA1V1 dKO), monotonic trends were additionally assessed using Spearman’s rank correlation, independently of group comparison tests.

All tests were two-tailed. A significance level of *p* < 0.05 was considered statistically significant. For multiple comparisons, significance was based on Holm-adjusted *p*-values.

## 3. Results

### 3.1. Quantitative Macroscopic Evaluation of Alcohol-Induced Gastric Mucosa Injury in Wild-Type and Knockout (TRPV1 KO, TRPA1 KO, TRPA1V1 dKO) Animals

**Scheme 1:**
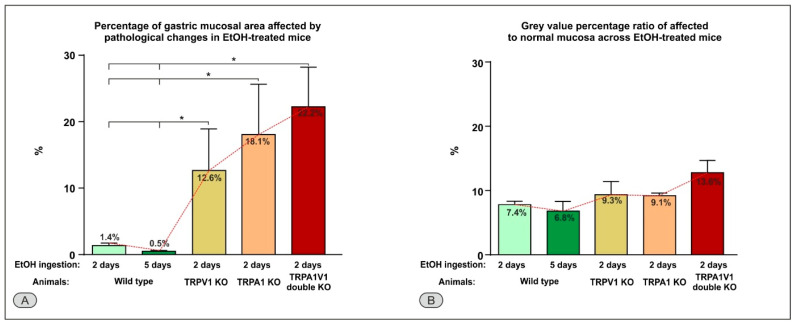
(**A**) Percentage of gastric mucosal area affected by pathological changes in ethanol-treated mice. ImageJ quantification of the pathologically affected surface area of the gastric mucosa. The mean ± SEM percentage of the affected area is shown for WT (2-day and 5-day alcohol exposure), *TRPV1* KO, *TRPA1* KO, and double KO (*TRPA1V1* dKO) mice. A significant increase in mucosal damage was observed in all knockout groups compared with WT controls (* *p* < 0.05, Mann–Whitney *U* test with Holm correction). The red line connecting group means illustrates the actual progression of values, showing a slight decrease between WT2 and WT5, followed by a marked increase in all knockout groups. Importantly, this graded progression from WT to double KO was statistically confirmed by Spearman’s rank correlation (ρ = 0.80, *p* < 0.0001), indicating a strong monotonic trend despite the local decrease in WT 5d. (**B**) Grey value percentage ratio of affected mucosa to normal mucosa across experimental groups. Bars show mean ± SEM for WT (2-day and 5-day EtOH), TRPV1 KO, TRPA1 KO, and TRPA1V1 double KO mice (*n* = 6 per group). The thin line connects group means to visualize the ordered progression (WT2 → WT5 → *TRPV1* KO → *TRPA1* KO → *TRPA1V1* dKO). The omnibus ANOVA did not indicate statistically significant differences between groups (F(4,25) = 2.62, *p* = 0.059). A weak monotonic increase across groups was observed in a separate trend analysis using Spearman’s rank correlation (ρ = 0.414, *p* = 0.023).

**Figure 1:**
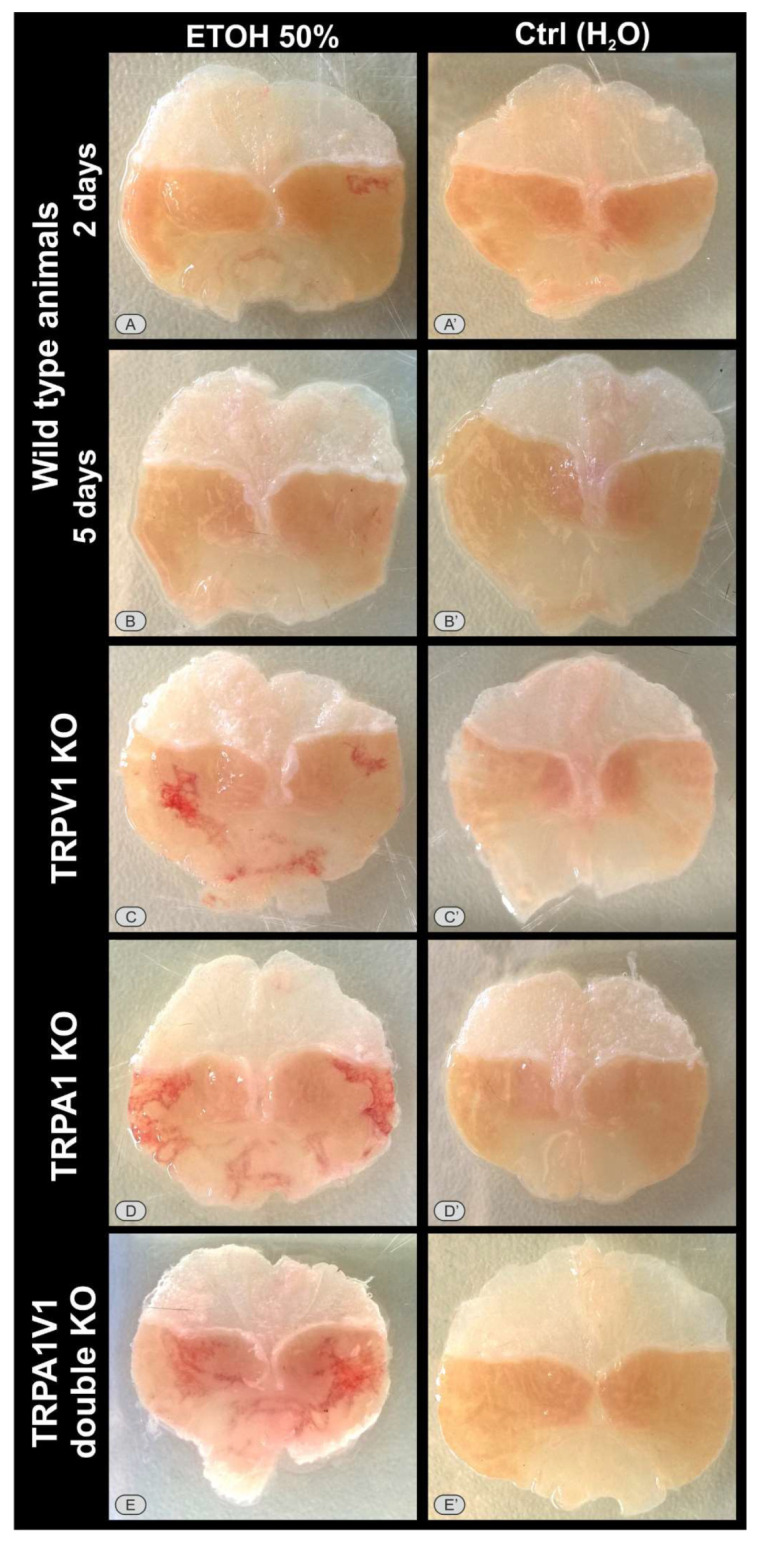
Macroscopic appearance of the mouse glandular stomach after ethanol exposure. Representative whole-mount images of gastric mucosa from wild-type (WT) and knockout (KO) mice after oral administration of 50% ethanol (EtOH; left column) or distilled water (Ctrl; right column). Rows: WT after 2 days (**A**,**A′**) and 5 days (**B**,**B′**); *TRPV1* KO (**C**,**C′**); *TRPA1* KO (**D**,**D′**); and double KO *TRPA1V1* (**E**,**E′**). Visible mucosal alterations are observed in EtOH-treated KO groups (**C**–**E**), whereas WT animals (**A**,**B**) show only limited changes. Water-treated controls display intact mucosa across all genotypes (**A′**–**E′**).

Macroscopic analysis of gastric tissue revealed that two days of ethanol administration caused significant changes to the mucosa of the knockout animals (Figure 1C–E), while in wild-type animals, these changes were barely observed in the second (Figure 1A) and fifth (Figure 1B) day of alcohol administration. Distilled water administration in controls of all animal groups had no damaging effect on the mucosa (Figure 1A′–E′). ImageJ analysis (Scheme 1A) revealed that the pathologically affected area of the glandular mucosa accounted for 12.6% in *TRPV1* KO (Figure 1C), 18.1% in *TRPA1* KO (Figure 1D), and 22.2% in *TRPA1V1* dKO (Figure 1E) animals. In wild-type animals, calculated values accounted for 1.4% and 0.5% after 2 (Figure 1A) and 5 (Figure 1B) days of EtOH administration, respectively (Scheme 1A).

Kruskal–Wallis analysis revealed highly significant differences between groups (*p* < 0.001). Post hoc Mann–Whitney *U* tests with Bonferroni correction showed that all knockout groups (*TRPV1* KO, *TRPA1* KO, and *TRPA1V1* dKO) exhibited significantly larger pathologically affected areas compared with WT controls (*p* < 0.05). However, no significant differences were observed between *TRPV1* KO, *TRPA1* KO, and *TRPA1V1* dKO (Scheme 1A). Nevertheless, a graded pattern of mucosal damage was evident, with *TRPA1V1* double KO animals showing the largest inflammatory areas, followed by *TRPA1* KO and *TRPV1* KO, while WT mice had the lowest values. This monotonic trend was confirmed by Spearman correlation (ρ = 0.80, *p* < 0.0001) (Scheme 1A).

Analysis of changes in the percentage of grayscale intensity in pathologically affected areas of the glandular stomach mucosa relative to normal mucosa showed the following values for *TRPV1* KO, *TRPA1* KO, and *TRPA1V1* double KO animals: 9.31%, 9.08%, and 13.61%, respectively (Scheme 1B). In wild-type animals, these values were lower—7.40% and 6.81% for animals administered EtOH for 2 and 5 days, respectively (Scheme 1B). After confirming normality (Shapiro–Wilk, all *p* > 0.05) and homoscedasticity (Levene’s test, *p* = 0.073), a one-way ANOVA indicated no statistically significant differences in grayscale percentages between groups (F(4, 25) = 2.62, *p* = 0.059). Therefore, these results should be interpreted with caution. In a separate analysis, a predefined ordered trend (WT2 → WT5 → *TRPV1* KO → *TRPA1* KO → *TRPA1V1* dKO) was evaluated using Spearman’s rank correlation, revealing a weak but statistically significant monotonic increase (ρ = 0.414, *p* = 0.023). A Tukey’s HSD post hoc test found no statistically significant pairwise differences between the groups.

### 3.2. Microscopic Evaluation of Alcohol-Induced Gastric Mucosa Injury in Wild-Type and Knockout (TRPV1 KO, TRPA1 KO, TRPA1V1 Double KO) Animals

**Figure 2:**
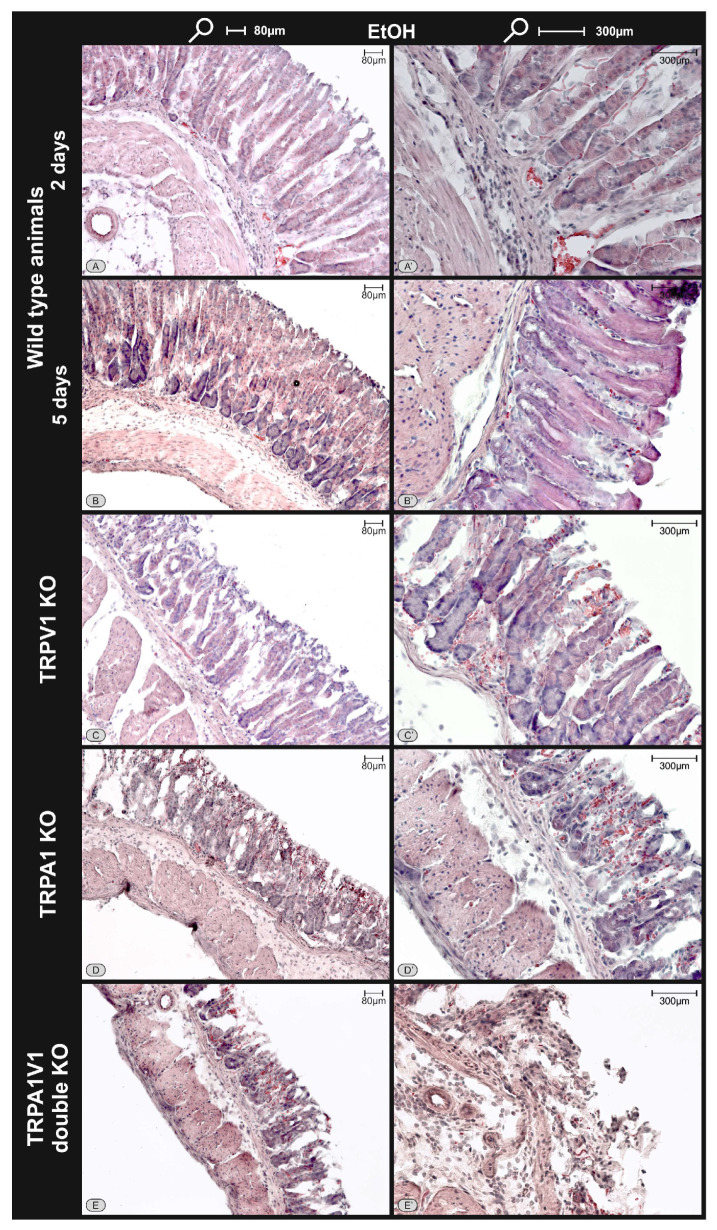
Ethanol-induced gastric mucosal injury in wild-type and TRP-deficient mice (H&E). Representative sections of the glandular stomach from wild-type mice treated with ethanol (EtOH) for 2 days (**A**,**A′**) or 5 days (**B**,**B′**), and from *TRPV1* KO (**C**,**C′**), *TRPA1* KO (**D**,**D′**), and *TRPA1V1* dKO (**E**,**E′**) animals. Panels with primes (**A′**–**E′**) show higher-magnification views. Histological features observed in EtOH-treated groups include submucosal widening consistent with edema, erythrocyte extravasation, and inflammatory-cell infiltration. Wild-type mice show minimal alterations, whereas epithelial changes such as sloughing and erosions are present in knockout animals. In *TRPA1V1* double knockout mice, the most extensive damage is observed, with lesions extending into deeper layers (**E**,**E′**). Water-treated controls showed no histopathological changes. Scale bars are indicated in the images.

Microscopic analysis of the stomach tissues stained with H&E revealed pathological changes in all animals receiving ethanol, while no changes were observed in control animals receiving water. Pathologies such as submucosal widenings due to edema, erythrocyte extravasations and inflammatory cell infiltration were observed in all ethanol groups (Figure 2). However, the degree of changes observed varied greatly between the studied animal groups: the slightest changes with occasional bleedings and low number of inflammatory cell infiltration occurred in wild-type animals after both 2 (Figure 2A,A′) and 5 (Figure 2B,B′) days of EtOH administration. Pathological changes observed in *TRPV1* KO (Figure 2C,C′) and *TRPA1* KO (Figure 2D,D′) mice were much more severe, with numerous areas characterized by focal epithelial cell sloughing and erosions, or even in occasional areas with almost total mucosal necrosis. Abundant extravasation of erythrocytes and a large number of inflammatory cells infiltrating both the mucosa and the submucosa were observed in numerous stomach areas in these animals.

The most extensive and severe pathological changes were observed in TRPA1V1 double KO animals (Figure 2E,E′), in which total mucosal necrosis involving the muscularis mucosae was observed in wide areas of the stomach wall, and voluminous submucosal widening due to massive edema was encountered. Areas adjacent to these severe lesions were characterized by abundant extravasation of erythrocytes and a large number of inflammatory cells infiltrating the mucosa, submucosa, and deep layers of the muscular layer.

### 3.3. Quantitative Real-Time PCR Evaluation of Expression Changes in Genes Encoding TRPA1, TRPV1 and Selected Proinflammatory Cytokines in Alcohol-Treated Gastric Tissues of Wild-Type and Knockout (TRPV1 KO, TRPA1 KO, TRPA1V1 Double KO) Animals

Real-time PCR analysis revealed changes in the expression of genes encoding *TRPA1*, *TRPV1* receptors and *IL-1β* and *IL-6* proinflammatory cytokines in the stomachs of alcohol-administrated animals (in relation to their corresponding H_2_O-treated controls), with values significantly differing between the different types of animals studied (wild-type animals, *TRPV1* KO, *TRPA1* KO, *TRPA1V1* dKO).

#### 3.3.1. TRPV1, TRPA1 Receptors

**Scheme 2:**
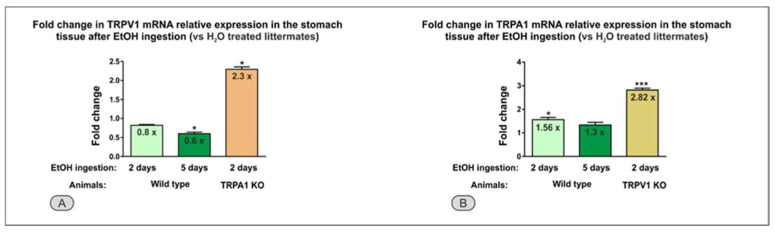
Effect of ethanol ingestion on gastric *TRPV1* and *TRPA1* mRNA expression. Bars show fold change relative to H_2_O-treated littermate controls (mean ± SEM). Asterisks denote statistical significance as indicated in the figure (* *p* < 0.05; *** *p* < 0.001; ns—not significant). (**A**) *TRPV1* mRNA: In wild-type mice, EtOH reduced *TRPV1* compared to H_2_O-treated littermates, reaching significance at day 5 (0.6×; day 2: 0.8×, ns). In *TRPA1* KO mice, EtOH increased *TRPV1* expression at day 2 (2.3×, *). (**B**) *TRPA1* mRNA: In wild-type mice, EtOH significantly increased *TRPA1* at day 2 (1.56×, *; day 5: 1.3×, ns). In *TRPV1* KO mice, EtOH markedly increased *TRPA1* expression at day 2 (2.82×, ***).

TRPV1 receptor in detail (Scheme 2A): In wild-type animals receiving alcohol, the expression of the *TRPV1* receptor was significantly reduced in relation to H_2_O-treated controls on the fifth day of the experiment and its fold change decrease accounted for 0.6, while after two days of EtOH ingestion, the decrease was not statistically significant. In *TRPA1* KO mice treated with EtOH, the *TRPV1* expression significantly increased, with a fold change of 2.29 in relation to H_2_O-administered littermate controls.

TRPA1 receptor in detail (Scheme 2B): The expression of *TRPA1* receptor mRNA was significantly increased in wild-type animals on the second day of EtOH administration, with a calculated fold change value of 1.56, while on the fifth day of the experiment, the change was not significant (in relation to H_2_O-administered controls). Administration of alcohol in *TRPV1* KO animals considerably increased *TRPA1* gastric expression, and its fold change was 2.82 in relation to their littermate H_2_O-administered controls.

#### 3.3.2. IL-1β, IL-6 Proinflammatory Cytokines

**Scheme 3:**
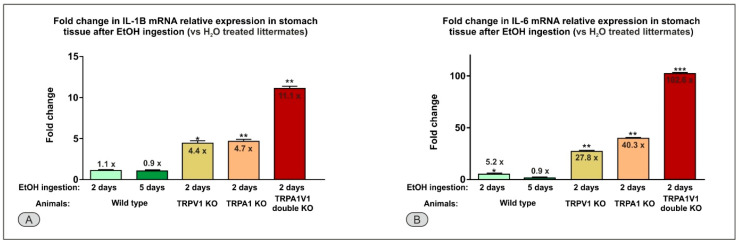
Effect of ethanol ingestion on gastric *IL-1β* and IL-6 *mRNA* expression. Bars show fold change relative to H_2_O-treated littermate controls (mean ± SEM). Asterisks denote statistical significance as indicated in the figure (* *p* < 0.05; ** *p* < 0.01; *** *p* < 0.001). (**A**) *IL-1β* mRNA: wild-type (WT) mice showed no significant change at days 2 or 5. After 2 days of EtOH, *IL-1β* increased significantly in *TRPV1* KO (4.4-fold), *TRPA1* KO (4.7-fold), and *TRPA1V1* dKO (11.1-fold) mice relative to H_2_O-treated littermates. (**B**) *IL-6* mRNA: wild-type (WT) mice showed a significant increase at day 2 (5.2-fold) but no significant change was observed on day 5. After 2 days of EtOH administration, *IL-6* was markedly elevated in all knockouts: *TRPV1* KO 27.8-fold, *TRPA1* KO 40.3-fold, and *TRPA1V1* dKO 102.6-fold increase vs. H_2_O-treated littermate controls.

In wild-type animals treated with EtOH, the *IL-1β* fold change expression (in relation to H_2_O-treated littermate mice) was not significantly altered on the second and fifth days of the experiment (Scheme 3A). In knockout animals treated with alcohol for two days, the *IL-1β* mRNA expression increased, and its fold changes (in relation to their littermate H_2_O-administered controls) were as follows: 4.4 for *TRPV1* KO, 4.7 for *TRPA1* KO and 11.1 for *TRPA1V1* double KO animals, and all these changes were statistically significant (Scheme 3A).

The fold change in *IL-6* mRNA expression (Scheme 3B) in wild-type animals was statistically significant on the second day of EtOH administration and amounted to 5.25, while in animals treated with ethanol for five consecutive days, the expression level value was similar to H_2_O-administered controls. In knockout animals treated with EtOH for 2 days, the values for *IL-6* expression significantly increased (in relation to their littermate H_2_O-administered controls), and the fold change for *TRPV1* KO was 27.82, for *TRPA1* KO it was 40.3, and for *TRPA1V1* double KO mice it reached the value of 102.56 (Scheme 3B).

### 3.4. Localization of TRPA1- and TRPV1-Immunoreactivity in the Gastric Wall

**Scheme 4:**
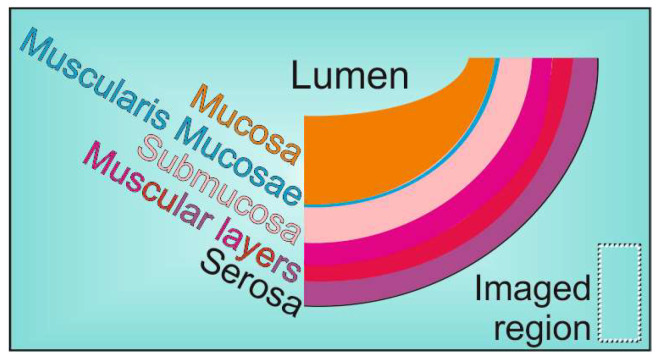
Schematic cross-section of the gastric wall used for anatomical orientation of the immunofluorescence micrographs. The scheme is also displayed in the corner of the micrographs to indicate the approximate location of the analyzed structures within the gastric wall.

Immunofluorescent staining combined with confocal microscopy illustrated the localization of TRPA1 and TRPV1 immunoreactive (TRPA1-IR, TRPV1-IR) structures within the stomach wall (Scheme 4) in wild-type control animals.

The purpose of this analysis was to provide anatomical context for the functional findings obtained in the knockout models, allowing for the identification of the gastric cell types that may contribute to receptor-dependent responses to ethanol.

Expression patterns of TRPA1 and TRPV1 immunofluorescence differed significantly. Both receptors were detected in nerve fibers within the gastric wall and in various other cell types located therein, such as enteroendocrine, chief, and parietal cells. Moreover, TRPA1-IR was also observed in myenteric neurons.

#### 3.4.1. Distribution of TRPV1-Immunoreactive Nerve Fibers in the Gastric Wall

**Figure 3:**
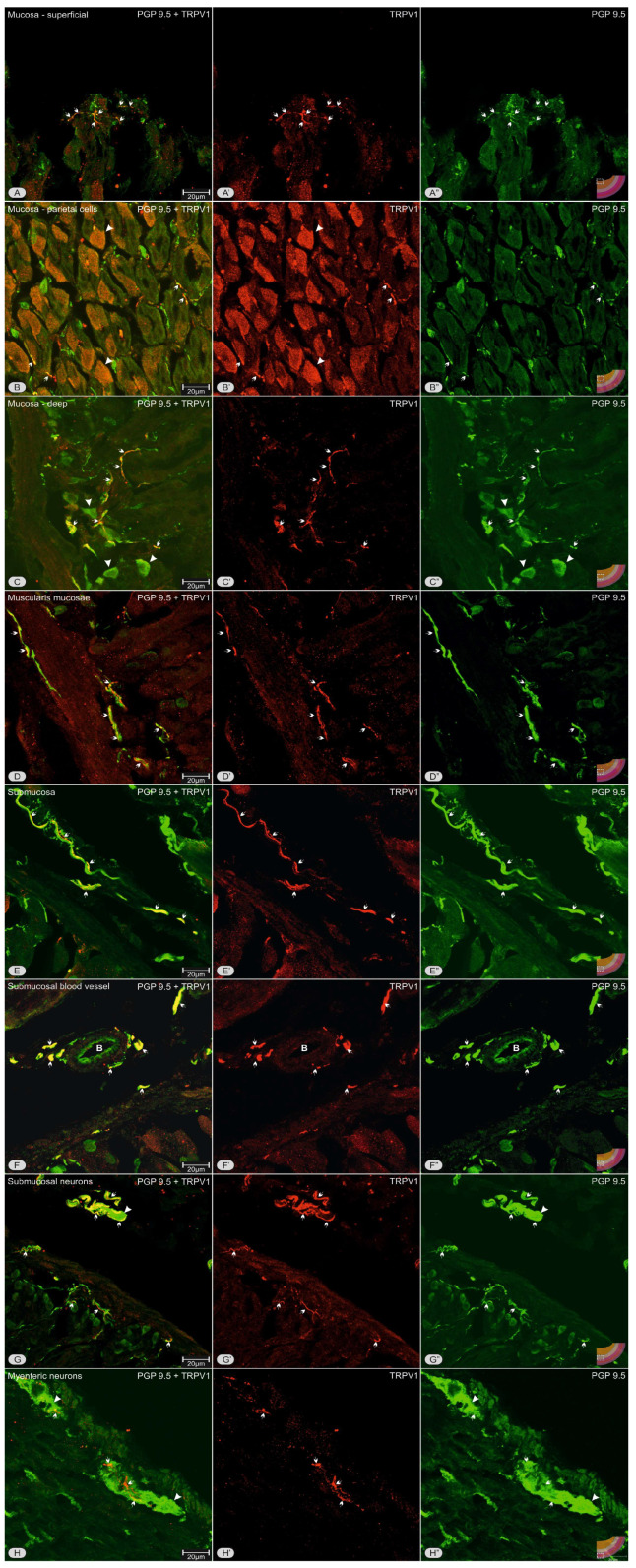
Nerve fibers double immunolabeled for PGP 9.5 and TRPV1 in stomach wall sections. Representative double immunofluorescence images of longitudinal gastric wall sections from control mice labeled for PGP 9.5 (green) and TRPV1 (red). The left column (**A**–**H**) shows merged images; the middle column (**A′**–**H′**) shows TRPV1 fluorescence; and the right column (**A″**–**H″**) shows PGP 9.5 fluorescence. Rows correspond to increasing tissue depth: superficial mucosa (**A**); mucosa at the level of parietal cells (**B**); deep mucosa in the pyloric antrum (**C**); muscularis mucosae (**D**); submucosa (**E**–**G**), which is shown in three complementary views to highlight: (**E**) nerve fibers dispersed in the submucosal connective tissue, (**F**) perivascular nerve fibers (the letter “B” denotes a blood vessel); and myenteric plexus (**H**). Arrows indicate nerve fibers double-labeled for PGP 9.5 and TRPV1. Arrowheads mark specific structures: in (**B**,**B′**), a TRPV1-immunoreactive parietal cell; in (**C**,**C″**), PGP 9.5-immunoreactive enteroendocrine cells; in (**G**,**G″**), the PGP-immunoreactive submucosal perikaryon; and in (**H**,**H″**), the PGP-immunoreactive myenteric neuron. Images are representative fields from different sections/areas (not serial sections). Scale bars in merged images should be considered the primary reference for image scaling.

**Figure 4:**
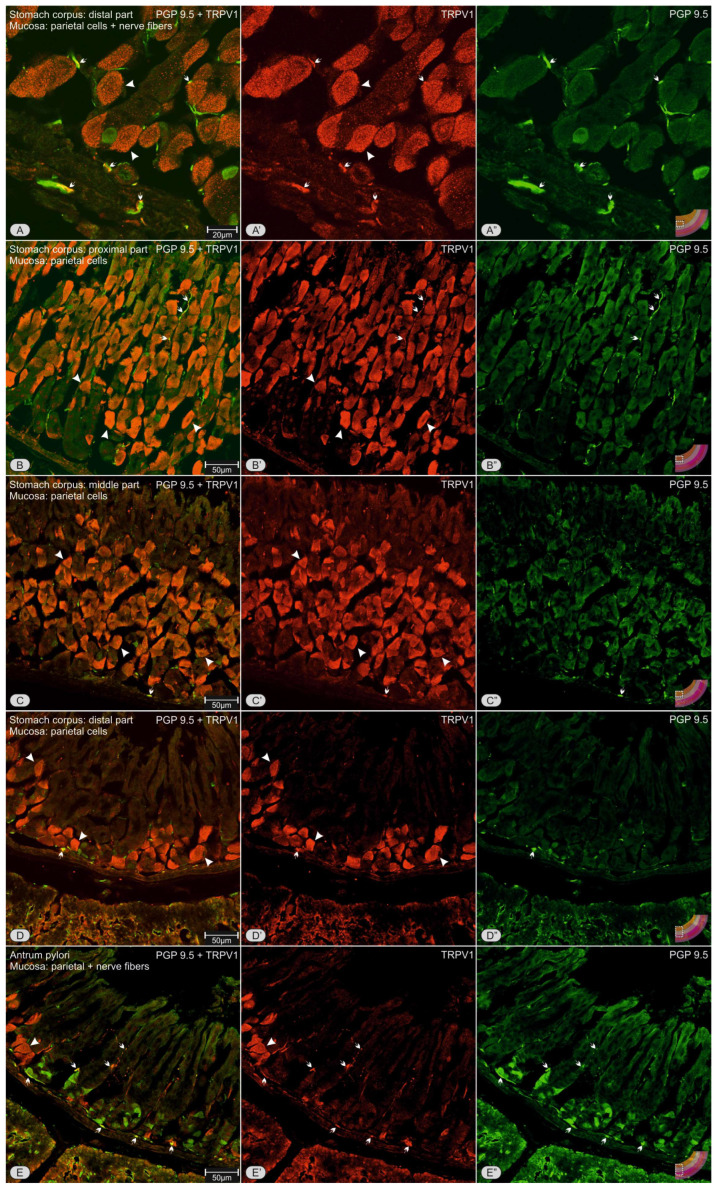
Stomach sections double immunolabeled for PGP 9.5 and TRPV1 showing TRPV1 immunoreactivity in gastric mucosal cells. Representative double immunofluorescence images of longitudinal stomach wall sections from control mice labeled for PGP 9.5 (green) and TRPV1 (red), showing glandular mucosa from different gastric regions (proximal/middle/distal corpus, and antrum pylori). The left column (**A**–**E**) shows merged images; the middle column (**A′**–**E′**) shows TRPV1 fluorescence; and the right column (**A″**–**E″**) shows PGP 9.5 fluorescence. Row (**A**–**A″**) presents a higher-magnification view of distal corpus mucosa. TRPV1 immunoreactivity is observed in mucosal cells consistent with parietal cell morphology. Arrowheads point to TRPV1 fluorescent parietal cells; arrows point to double immunolabeled (PGP 9.5-/TRPV1-immunoreactive) nerve fibers. Scale bars in merged images should be considered the primary reference for image scaling.

TRPV1 immunofluorescent nerve fibers were observed across the entire mucosa thickness (Figure 3A–G), starting from the most superficial layers, where TRPV1-IF neuronal fibers bordered the surface epithelial cells, and in some locations even formed a kind of nerve fiber cluster (Figure 3A,A′,A″). Most of these fibers were noticed in the distal stomach. Towards the deeper mucosa, i.e., around the middle depth mucosa layer, the fibers were much less frequently observed; however, they were present even in the proximal part of the stomach, that is, in the fundic part of the oxyntic mucosa (Figure 3B,B′,B″). In the deepest parts of the mucosa (Figure 3C,C′,C″; Figure 4A,A′,A″; Figure 4C,C′,C″; Figure 4D,D′,D″; Figure 4E,E′,E″) as well as in the muscularis mucosa (Figure 3D,D′,D″) and submucosa (Figure 3E,E′,E″) prominent nerve fibers were observed, mainly in the distal stomach. Moreover, some TRPV1-IR neuronal fibers were encountered in the vicinity of the submucosal blood vessels (Figure 3F,F′,F″) as well as accompanying occasional submucosal perikarya (Figure 3G,G′,G″). Analysis of deeper parts of the muscular layers revealed TRPV1-IR in nerve fibers adjacent to some myenteric perikarya (Figure 3H,H′,H″).

#### 3.4.2. TRPV1-Immunoreactive Mucosal Cells

TRPV1 fluorescence was observed in a large number of cells whose morphology, localization, and spatial arrangement (the majority observed in the fundus) matched that of parietal cells (Figure 4). These cells were characterized by medium to strong, sand-like immunofluorescence evenly dispersed along the cell body (Figure 4A,A′,A″). The highest number of cells was observed in the proximal stomach, i.e., in the gastric fundus (Figure 4B,B′,B″), and towards the distal stomach, their number gradually decreased (Figure 4C,C′,C″,D,D′,D″) reaching a minimum at the border with the pyloric antrum (Figure 4E,E′,E″), while they were not observed in the pyloric mucosa. As depicted earlier, occasional PGP 9.5/TRPV1 double-immunoreactive nerve fibers were captured in the vicinity of parietal cells, while thick bundles of nerve fibers and varicosities were occasionally observed in the deep layers of the distal stomach (Figure 4A,A′,A″) and pyloric mucosa (Figure 4E,E′,E″).

#### 3.4.3. TRPA1-Immunoreactive Nerve Structures

**Figure 5:**
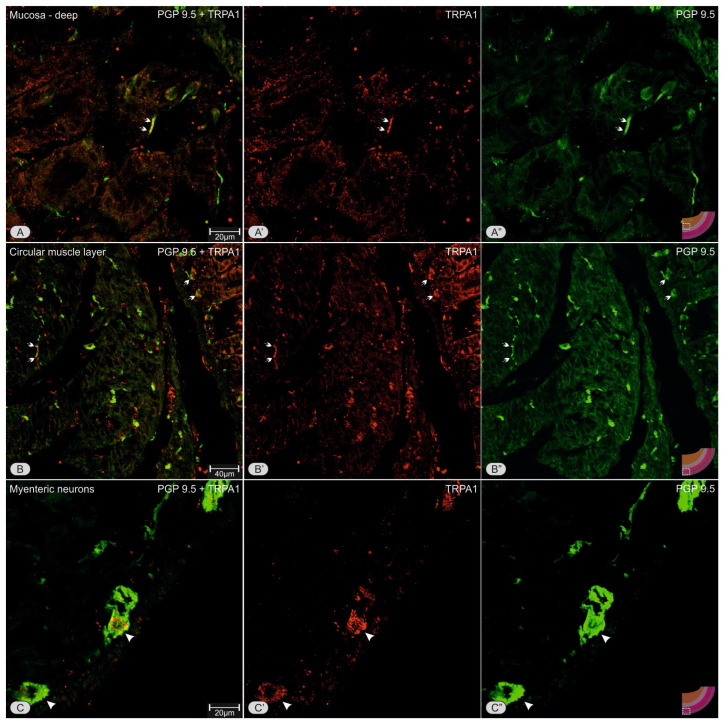
Nerve structures co-expressing PGP 9.5 and TRPA1 in the mouse gastric wall. Representative dual immunofluorescence images of longitudinal gastric wall sections from control mice labeled for PGP 9.5 (green) and TRPA1 (red). Panels are arranged in three columns: merged channels (**A**–**C**), TRPA1 fluorescence (**A′**–**C′**), and PGP 9.5 fluorescence (**A″**–**C″**). Rows correspond to deep mucosa (**A**–**A″**), circular muscle layer (**B**–**B″**), and myenteric plexus at the interface between circular and longitudinal muscle layers (**C**–**C″**). TRPA1 immunoreactivity is sparse and observed mainly in isolated nerve fibers within the deep mucosa (**A**–**A″**) and circular muscle layer (**B**–**B″**). Occasional TRPA1-immunoreactive myenteric neuronal perikarya (arrowheads) are also present (**C**–**C″**). Arrows denote nerve fibers double-labeled for PGP 9.5/TRPA1 when present. Images are representative fields from different sections/areas (not serial sections). Scale bars in merged images should be considered the primary reference for image scaling.

**Figure 6:**
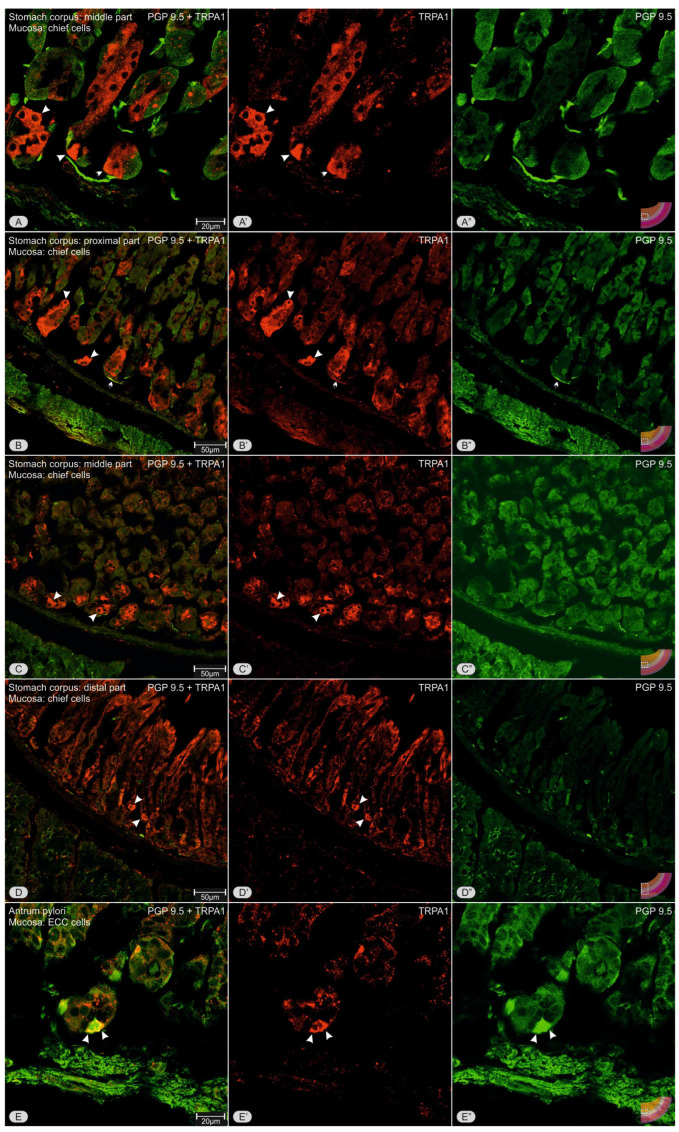
TRPA1 immunoreactivity in gastric mucosal cells of the mouse stomach. Representative double immunofluorescence images of longitudinal stomach sections from control mice labeled for PGP 9.5 (green) and TRPA1 (red). Panels are arranged in three columns: merged images (**A**–**E**), TRPA1 fluorescence (**A′**–**E′**), and PGP 9.5 fluorescence (**A″**–**E″**). Rows depict different regions of the gastric mucosa, including proximal (**B**–**B″**), middle (**C**–**C″**), and distal corpus (**D**–**D″**), and pyloric antrum (**E**–**E″**). Panel (**A**–**A″**) shows a higher-magnification view of the corpus mucosa. TRPA1 immunoreactivity is observed in mucosal cells located within the deep gastric glands, consistent with chief cell morphology (arrowheads in panels (**A**–**D; A′**–**D′**)). Occasional TRPA1-positive cells consistent with enteroendocrine cells are also present in the distal gastric mucosa (arrowheads in panel (**E**–**E”**)). Arrows indicate nerve fibers double-labeled for PGP 9.5 and TRPA1. Images represent fields from different regions and are not serial sections. Scale bars in merged images should be considered the primary reference for image scaling.

TRPA1-IR tiny, extremely rare, singular nerve fibers were noticed in the deepest layers of the mucosa (Figure 5A,A′,A″ and Figure 6B,B′,B″) and within the muscular layer (Figure 5B,B′,B″); however, they were dispersed randomly, and no characteristic occurrence was observed depending on the stomach part.

Microscopic analysis of neuronal cell bodies revealed that some of the myenteric perikarya were TRPA1-immunofluorescent (Figure 5C,C′,C″).

#### 3.4.4. TRPA1-Immunoreactive Mucosal Cells

Medium to strong, highly grainy TRPA1 fluorescence was observed in cells of the deep mucosa (Figure 6A,A′,A″), which morphology, localization and spatial arrangement closely resembled chief cells. These cells were observed along the fundic mucosa, in its proximal (Figure 6B,B′,B″), middle (Figure 6C,C′,C″) and distal (Figure 6D,D′,D″) parts.

Additionally, singular or small groups of intensely TRPA1-immunofluorescent cells were encountered in the deep layers of the distal stomach mucosa, whose localization and morphology corresponded to enteroendocrine cells (Figure 6E,E′,E″). The TRPA1 immunofluorescent precipitate formed characteristic clamp- or sand-like highly fluorescent inclusions within the positive cells.

### 3.5. Specificity Control Staining

**Figure 7:**
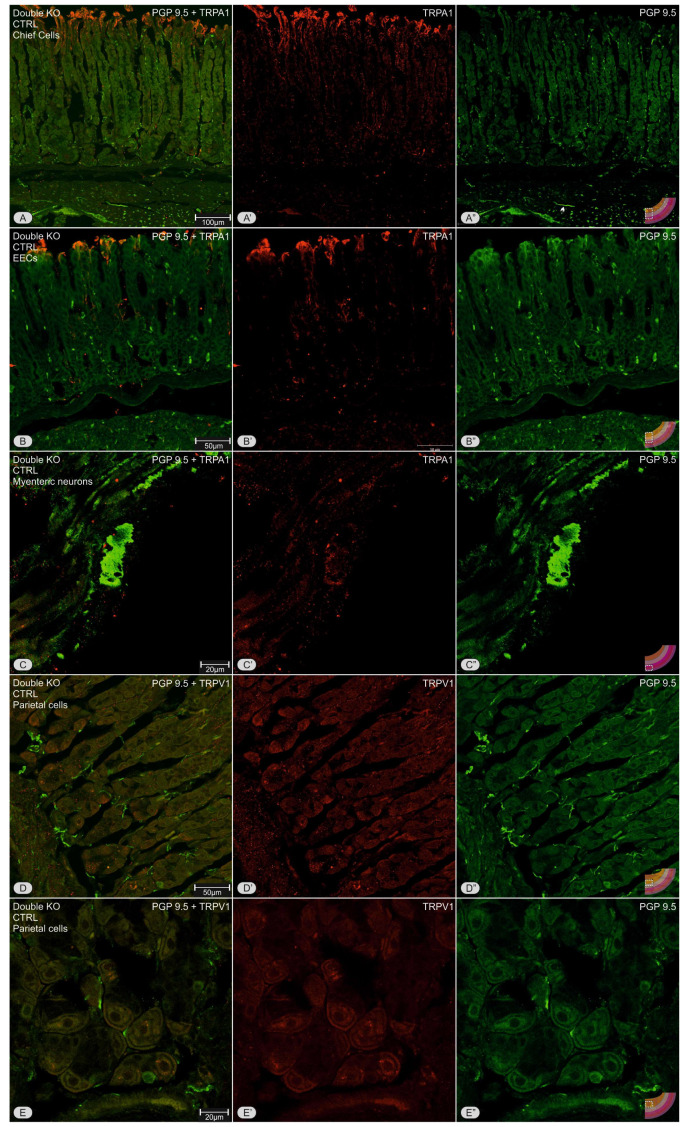
Antibody specificity control staining in the TRPA1/TRPV1 double-knockout mouse gastric wall. Representative dual immunofluorescence images of longitudinal gastric wall sections from *TRPA1/TRPV1* double-knockout mice labeled for PGP 9.5 (green; (**A″**–**E″**)) and either TRPA1 (red; (**A**,**A′**,**B**,**B′**,**C**,**C′**)) or TRPV1 (red; (**D**,**D′**,**E**,**E′**)). Panels are arranged in three columns: merged images (left), red channel (middle), and PGP 9.5 fluorescence (right). PGP 9.5 labeling is preserved, indicating neuronal structures, while no specific TRPA1 or TRPV1 immunoreactivity is detected in the knockout tissue. Background fluorescence is present in the red channel, including diffuse and punctate signals within mucosal and submucosal regions. Rows (**A**,**A′**,**B**,**B′**) show a high-intensity superficial signal at the luminal edge and low-intensity fine granular staining; both are considered non-specific and were excluded from analysis. Occasional dot-like and clumpy background signals are also observed within mucosal cells (**D**,**D′**,**E**,**E′**). In contrast, myenteric neuron cell bodies (**C**,**C′**,**C″**) show strong PGP 9.5 labeling with minimal red background. These control images were used as a reference for distinguishing specific from non-specific immunofluorescence in experimental sections. Arrow shows PGP 9.5 immunoreactive nerve fiber (**A″**). Images represent fields from different regions and are not serial sections. Scale bars in merged images should be considered the primary reference for image scaling.

High-power confocal laser microscope scans revealed some nonspecific background fluorescence scattered throughout tissue samples immunolabeled with anti-TRPA1 and anti-TRPV1 antibodies, as depicted by additional control staining using TRPA1V1 dKO animal tissues (Figure 7).

These nonspecific TRPA1-immunoreactive structures mostly appeared as highly fluorescent, irregularly dispersed, and clearly visible dot-like or irregularly shaped formations (Figure 7A,A′,B,B′). They also occurred as sand-like or tiny fluorescent particles, both within the tissue and inside myenteric plexus perikarya (Figure 7C,C′,C″). Moreover, a strong nonspecific TRPA1-immunofluorescent signal was detected in the superficial linear layer of mucosal surface cells, within the mucus, and along some glandular tubules (Figure 7A,A′,B,B′).

A nonspecific TRPV1 fluorescent signal occurring as tiny to medium sand-like particles and dot-like structures was observed mainly in the mucosa and muscularis mucosa layer (Figure 7D,D′). Moreover, some dot- and clumpy-like nonspecific TRPV1 immunoreactivities were observed within the mucosa oxyntic cells (Figure 7D,D′,E,E′).

However, it should be underlined that the comparison of double immunolabeled tissue scans obtained from both TRPA1V1 dKO and wild-type animals, with consideration of the specific pattern of each immunofluorescence type, allowed for a high probability distinction between the truly positive immunostaining and false positive background.

## 4. Discussion

Our results revealed the role of both studied TRP receptors in gastric tissue’s reaction to ethanol and showed differences in their involvement based on receptor type and co-occurrence. Moreover, we demonstrated changes in both receptors’ mRNA expression in gastric tissues after alcohol administration in wild-type animals and in relevant knockouts, as well as verified time-dependent changes (for wild-type animals), indicating the adaptive reaction of gastric tissues to alcohol intake. Finally, we illustrated the localization of both receptors in the stomach wall tissue.

Taken together, the functional data obtained in the knockout models, combined with the cellular localization of TRPA1 and TRPV1, suggest that these receptors may contribute to gastric mucosal protection through both neuronal and non-neuronal mechanisms within the gastric wall.

We applied an ethanol-induced gastric lesion model using 50% EtOH administered by intragastric gavage because this technique allows for precise application of the disrupting reagent, ensures reproducibility and triggers a rapid inflammatory response. Moreover, this method affects only the stomach tissue without involving other intestinal sections, thus preventing the influence of other “non-gastric” factors [30]. Additionally, as ethanol is a common component of beverages consumed by humans, the findings may have clinical relevance.

### 4.1. Ethanol-Induced Changes in Wild-Type Animals—Proinflammatory Interleukins and TRP Receptors

Ethanol administration in wild-type animals resulted in minimal pathological changes in the gastric mucosa after both 2 and 5 days of exposure, with almost no significant macroscopic or microscopic differences in mucosal morphology. The unchanged level of mRNA encoding *interleukin-1β*, which typically shows an early and transient increase during inflammation, indicates that ethanol caused only a mild inflammatory response that had already resolved by the time of measurement. However, variations in mRNA expression encoding *IL-6* were more obvious and differed between the second and fifth days of the experiment. On the second day of alcohol administration, *IL6* mRNA expression increased, while on the fifth day, fold-change values were reduced to nearly baseline levels. These results suggest that wild-type animals possess defense mechanisms that regulate inflammatory processes to protect the gastric mucosa against ethanol exposure. The time-dependent reduction in interleukin expression indicates adaptive changes in the stomach tissue that prevent uncontrolled gastritis.

Interestingly, ethanol administration led to a gradual downregulation of *TRPV1* mRNA in WT animals, which decreased over time. This mechanism, combined with minor macroscopically and microscopically observed mucosal lesions and decreasing mRNA levels of proinflammatory cytokines, suggests that gastric wall cells intentionally and gradually reduce receptor expression. Since the TRPV1 receptor is activated by both HCL and ethanol, it can be assumed that these are adaptive changes against the continuous stimulation of gastric cells due to prolonged exposure to alcohol. The gastric reaction to ethanol as well as the protective function of the introduced capsaicin were observed just after 30 min after the EtOH/capsaicin administration [35]. Therefore, after 2 and 5 days of alcohol stimulation, the adaptive reduction of *TRPV1* expression seems to be a reasonable reaction against prolonged irritation. In fact, the reduced expression of *TRPV1* in wild-type animals after alcohol administration may be a way to reduce the excitability of these receptors under multiple activating factors. Although stomach function and tissue structure differ from those of the intestines, our results are also consistent with data from tissue biopsies of patients with active ulcerative colitis, in which decreased *TRPV1* expression was observed [17]. Remarkably, in the colon of dextran sulfate sodium (DSS)-administrated mice, as shown in the same study, changes in *TRPV1* expression were not significant compared to healthy animals, but receptor expression was reduced in a time-dependent manner, similar to the present experiment.

Interestingly, in the indomethacin-induced gastritis [36], changes in *TRPV1* mRNA expression were completely opposite to our results, with levels significantly increased in the stomach wall under pathological conditions. These disparate results suggest completely different mechanisms of TRPV1 receptor action in the gastric reaction to alcohol and iodoacetamide. This is consistent with contradictory data on the role of TRPV1/TRPA1 in different colitis models, where their involvement appears to be strictly dependent on the type of key inflammatory pathomechanisms [13,14,15,16]. Both chemical agents affect the mucosa differently. Iodoacetamide, a water-soluble sulfhydryl-alkylating chemical, depletes sulfhydryl groups, including the protective antioxidant glutathione (GSH) in the cells, facilitating reactive oxygen species production and gradual oxidative tissue damage [37]. By contrast, ethanol-induced mucosal lesions develop very rapidly, because EtOH impairs mucus synthesis and secretion and damages epithelial cells, exposing the underlying mucosa to alcohol and gastric luminal acid [38].

Changes in *TRPA1* receptor expression in wild-type animals, similar to *TRPV1*, revealed their reaction and time-dependent fluctuations to alcohol intake; however, important differences were observed. Alcohol administration resulted in an overall increase in *TRPA* expression in the stomach tissue, which was opposite to the reduced expression of *TRPV1*, but the time-dependent tendency to decrease expression was similar for both receptors.

Our results on *TRPA1* increased expression and time-dependent changes under alcohol gastritis in wild-type animals are congruent with data presented in iodoacetamide-evoked gastritis, in which its elevated expression decreased along the studied timepoints [36]. Such congruent data indicate that the tissue reaction of gastric TRPA1 receptors is similar for both pathological factors.

Moreover, the obtained results on TRPA1 increase are in line with data showing elevated TRPA1 expression in intestinal tissue biopsies from patients with Ulcerative Colitis and Crohn’s disease [13,17] as well as its increased expression observed in dorsal root ganglia under DSS-evoked colitis [39]. Although our results revealed time-dependent changes in *TRPA1* expression, indicating adaptive alterations of the gastric wall, the sustained elevation of its level at both examined time points indicates ethanol-induced upregulation of the receptor. Such observations clearly suggest a protective role of TRPA1 in the gastric mucosa against various disruptive factors, including EtOH-induced irritation.

### 4.2. Impact of TRPV1 and TRPA1 Deficiency on Gastric Mucosa Reaction to Ethanol

Our results obtained in the groups of knockout animals confirmed some gastroprotective and anti-inflammatory roles of TRPV1 for ingested ethanol. Unlike WT, *TRPV1* KO mice exhibited increased expression of *TRPA1*, suggesting a compensatory mechanism attempting to counteract the absence of TRPV1. In line with our observations, TRPV1 was also suggested to protect against inflammatory conditions in gastrointestinal tract experiments [3,7,8,40]. However, completely opposite results, indicating a pro-inflammatory function of TRPV1, were obtained in indomethacin-induced gastric ulcerations in *TRPV1* KO animals [10]. These findings once again highlight completely the different mechanisms of gastric mucosal lesions in indomethacin-induced gastritis and emphasize the need to study different pathological conditions separately.

The protective role of capsaicin on gastrointestinal mucosa is commonly linked with TRPV1-dependent sensory neurons activation [25,41]. According to such “TRPV1-neural sensory-dependent” theory, we can speculate that TRPV1 receptor-deficient neurons in *TRPV1* KO and *TRPA1V1* double KO animals were not properly activated by infused alcohol; thus, they were unable to react accurately and did not release regulatory neuropeptides (such as CGRP and SP) from their gastric nerve endings [24,25]. Since both TRPV1 and TRPA1 function as non-selective cation channels, their activation leads to Ca^2+^ influx and neuronal excitation, which constitutes a key mechanism underlying TRP receptor-mediated cellular responses, triggering neuropeptide release [42]. CGRP is known to promote vasodilation, increase mucosal blood flow, and support protective mechanisms within the gastric wall [43]. Therefore, the increased susceptibility to ethanol-induced injury observed in receptor-deficient animals may, at least in part, reflect impaired neuropeptide-mediated protective signaling. These observations are consistent with previous reports indicating that TRP receptors play an important role in the regulation of mucosal defense and inflammatory responses in the gastrointestinal tract [25,44,45].

As a natural consequence, the mucosal reaction observed in knockout mice was not precisely controlled by neuropeptides, as it is in wild-type animals. However, it should be mentioned that most studies focused on TRPV1-dependent sensory nerve reactions applied large doses of injected capsaicin as a technique for chemical deafferentation [25,46]. Importantly, in such experiments the administered capsaicin affected the whole organism and must have also influenced other gastric structures containing TRPV1 receptors; thus, their physiology must have also been affected. Observations on the influence of capsaicin on gastrointestinal cell cultures devoid of any neuronal supply [47,48] clearly demonstrated that cells other than neuronal cells are also highly vulnerable to capsaicin treatment. Our and other studies [49,50,51] revealed the presence of the TRPV1 receptor in structures other than sensory nerve fibers, such as in parietal cells. In line with this, piperine, a TRPV1 activator with much greater receptor efficiency than capsaicin, significantly reduced the volume and acid output in pyloric-ligated rats, functionally indicating a relation between parietal cells and TRPV1 [3]. Moreover, PCR measurements revealed the presence of mRNA encoding *TRPV1* in the stomach wall [36,52] and additionally, its tissue expression was significantly influenced by gastritis [36]. Sensory nerve cell bodies supplying the stomach occupy the dorsal root ganglia and vagal nodose ganglia [53,54], and these structures are the main source of mRNA related with extrinsic sensory nerve supply and are also influenced by factors disrupting the stomach mucosa [55]. In contrast, the majority of mRNA extracted from the stomach wall tissue (as in our and other experiments) is derived from the cell bodies located therein (such as stomach mucosa cells).

It should be noticed that influx and resident immune cells expressing the TRPV1 receptor may be another source of mRNA extracted from the stomach tissue [56]. Thus, gastric cells other than sensory neurons should also be considered as potentially involved in the TRPV1-dependent gastric response to ethanol and studied in the future.

However, the localization of TRPA1 and TRPV1 immunoreactivity in non-neuronal gastric cells observed in the present study suggests that neurogenic mechanisms may not be the only components involved. Ethanol-induced gastric injury is associated with local epithelial responses, including disruption of the mucosal barrier, inflammatory signaling, and adaptive cellular processes. Thus, TRP receptor-dependent protection may involve both sensory-neuronal and local mucosal mechanisms within the gastric wall. This is in line with previous reports indicating that TRP receptors are also expressed in non-neuronal cell types, including epithelial cells, and may participate in local regulatory processes within tissues [57].

Linking the present findings on TRPV1 localization and expression in the gastric wall with detailed knowledge of TRPV1 receptor activation patterns [58,59,60], it seems reasonable to predict that these receptors may exhibit some unique properties in the stomach. Alcohol activation of the receptor in the acidic environment dramatically decreases the threshold of its temperature activation [60,61]. In agreement, many opinions suggest that the burning sensation accompanying the consumption of alcohol results from the activation of TRPV1 receptors in the mucosa of the upper gastrointestinal tract [61]. Importantly, the pH in the stomach is highly acidic under physiological conditions (it can even drop to pH 1 after a high-protein meal); thus, TRPV1 sensory nerve fibers deeply penetrating the mucosa, along with all the other stomach wall cells expressing TRPV1 (as observed in the present study), would be extremely vulnerable to activation under any reduction or even slight damage to the protective mucus layer. Since there are results indicating differences in expression, functionality and conditions of thermal stimulation between genetic variants of the TRPA1 protein [62], which belongs to the same receptor family, we suggest the need for future physiological studies aimed at understanding the function and relation to HCL of gastric wall TRPV1 receptors, which can demonstrate some unique properties.

An extremely interesting pattern was observed in *TRPA1* KO animals, where the stomach mucosa response was even stronger than in *TRPV1* KO, despite TRPA1 often being described as pro-inflammatory [63,64,65]. Most studies relate such proinflammatory reactions with the activation of TRPA1 in sensory afferent nerves and the subsequent release of neuropeptides that trigger neurogenic inflammation [66,67]. The highest *IL-1β* and *IL-6* upregulation among single knockouts was observed in this group, along with severe mucosal area expansion and a non-significant tendency toward increased grayscale differences. This may indicate that TRPA1 contributes even more strongly to gastric mucosal protection than TRPV1 in ethanol-induced gastric mucosa pathology. Although both receptors are expressed on CD4+ T lymphocytes, TRPA1 inhibits TRPV1 activity in these cells, limiting inflammatory responses [13]. The absence of TRPA1 may have removed this inhibitory effect, allowing TRPV1 to remain hyperactivated, further exacerbating the pathological reaction. In line with this, TRPA1 is known to play a protective role in colitis by inhibiting the activation of CD4+ T cells, which is directly associated with TRPV1 receptors [13].

Comparing these findings to other models, TRPA1 has shown both pro- and anti-inflammatory effects depending on the context. For example, its desensitization with cannabidivarin reduced intestinal inflammation by lowering immune cell infiltration and cytokine production [68]. Additionally, TRPA1 activation in DSS-induced colitis models has been shown to decrease pro-inflammatory cytokines, while *TRPA1* KO worsened inflammation [17]. Thus, our findings support an anti-inflammatory and highly protective role for TRPA1 in gastric mucosa, consistent with intestinal models.

Further support for a potential protective role of TRPA1 may be derived from its localization in gastric wall cells. Our confocal laser microscope analysis revealed TRPA1 immunoreactivity in structures resembling chief cells, neuroendocrine cells, and occasional myenteric neurons. However, given the relatively weak and sparse signal, particularly in neuronal structures, these findings should be interpreted with caution. The results demonstrating the high expression level of mRNA encoding TRPA1 in the human, mouse and rat stomach tissues [69], as well as its expression changes observed in the stomach under gastritis (present study and [36]), support the presence of the TRPA1 receptor on cells located directly in the stomach wall, not only on extrinsic sensory nerve fibers. Based on these observations, it is possible that some of these TRPA1 immunoreactive enteroendocrine cells observed in the antrum pylori, depending on their specific type, may release somatostatin (G cells), gastrin (G cells) or serotonin (EC cells), which may contribute to mucosal protective mechanisms against ethanol and could be affected by the absence of *TRPA1* in knockout animals. Although these interpretations remain speculative and require functional validation, they are consistent with reports that enterochromaffin cells, which are known to strongly express TRPA1, release serotonin, affecting motility and gastric protection [69]. Similarly, the presence of TRPA1 immunoreactivity in cells resembling chief cells may suggest a possible role in zymogen secretion; however, its absence in KO animals may be associated with altered gastric homeostasis and the observed mucosal changes. Finally, the occasional presence of TRPA1 in myenteric plexus structures may suggest a potential involvement in the regulation of gastric motor activity. The absence of TRPA1 in knockout animals could therefore be associated with altered motility and related changes in gastric function. In line with this, TRPA1-immunoreactivity was observed in the myenteric ganglia of the stomach antrum, cecum and colon [70]. Altogether, the present findings suggest that TRPA1 may be present not only in extrinsic sensory neurons but also in intramural gastric cells. This may indicate a potential role for TRPA1 in ethanol-induced gastric protection, extending its function beyond extrinsic sensory pathways to include intramural cells involved in mucosal defense. However, these functional implications require further experimental validation. Future research could focus on characterizing the distinct roles of these intramural TRPA1-expressing cells in various physiological processes, particularly those dependent on or modulated by TRPA1 activity.

It is important to note that immunocytochemical staining of both receptors should be approached with caution, as the method carries inherent limitations in such applications. The weak, unspecific background signal observed in knockout animals may result from the fact that some target sequences recognized by the antibodies applied might be conserved in the knockout animals, in which the receptors’ functionality was abolished [63,71]. A similar situation was observed in the detailed immunofluorescent studies on the TRPA1 localization within the gastrointestinal tract [70]. Moreover, these authors also observed an unspecific pattern for standard control staining, which can additionally suggest some unique cross-reactivity properties of TRPA1 epitopes. In respect to TRPV1 immunofluorescence, the bioinformatic analysis revealed minor similarity of the TRPV1 amino acid sequence (NP_001001445) to mCG4972 (EDL14029.1) and potassium voltage-gated channel subfamily H (XP_036016336.1). Accordingly, Heitzman states that K channels are a prerequisite for the production of hydrochloric acid [72]. Thus, the partial overexpression of the signal visualized by the antibody cannot be excluded.

### 4.3. The Cumulative Impact in Double Knockout (TRPA1V1 dKO) Mice

The most pronounced tissue response to ingested ethanol was observed in *TRPA1V1* dKO animals. This was confirmed across all assessment methods, including pathologically disrupted mucosal area analysis, grayscale evaluation, and the highest *IL-1β* and *IL-6* expression levels. Importantly, our study revealed increased expression of the *TRPV1* receptor in *TRPA1* KO animals and the *TRPA1* receptor in *TRPV1* KO animals, indicating a natural attempt of the stomach cells to compensate for their deficiencies by using a second type of receptor. The lack of compensatory mechanisms in double *TRPA1V1* knockdown animals may explain the development of the strongest pathological stomach reaction in this animal group.

Some insight into the pronounced alterations observed in the double knockout animals can be gained from our confocal microscopy analysis of the gastric wall. As previously mentioned, our analysis revealed distinct, non-overlapping cellular populations expressing TRPA1 and TRPV1, including enteroendocrine, parietal, chief cells and myenteric neurons. While TRPV1 and TRPA1 were detected in different subsets of cells, the simultaneous loss of both receptors in the double KO animals led to widespread dysfunction across these populations. Moreover, not only were cell-specific functions probably disrupted, but also any potential compensatory interactions between TRPA1 and TRPV1 were eliminated in this group of animals.

The fact that both receptors are expressed on CD4+ T cells and that their interaction precisely regulates the activation of immune cells [13] strongly suggests that impaired immune regulation and the reduction of possible compensatory mechanisms in double TRPA1V1 knockouts may contribute to the altered gastric defense responses observed in these animals.

Additionally, complete depletion of both TRPA1 and TRPV1 receptors must have significantly impaired the function of extrinsic sensory neurons, disrupting the well-established and commonly accepted neuroinflammatory regulatory pathways at multiple levels of the neuronal system. As a result, the antidromic release of gastroprotective neuropeptides from peripheral nerve endings, widely recognized as key mechanisms in gastric mucosal defense, was effectively abolished. This functional paralysis of the sensory afferent system is further supported by findings indicating that TRPA1 and TRPV1 can form heteromeric channel complexes in sensory neurons, contributing to their unique biophysical properties [64,73]. In double knockout animals, the absence of such complexes may further exacerbate mucosal vulnerability and promote uncontrolled inflammatory responses in the gastric environment.

All our observations and related considerations are consistent with previous predictions that the simultaneous deletion of both receptors—members of the same TRP family, often co-expressed in neuronal structures, acting synergistically and exerting mutual regulatory effects—would result in the most pronounced pathological response. Importantly, these findings also confirm that both TRPA1 and TRPV1 play protective roles in the gastric mucosa under ethanol-induced stress, and their presence is crucial for maintaining gastric integrity and homeostasis.

### 4.4. Water-Treated Knockout (TRPA1 KO, TRPV1 KO, TRPA1V1 dKO) Mice

An interesting observation arises from the results obtained in control animals across all knockout groups. The absence of any pathological changes following water administration clearly indicates that, despite the genetic deletion of individual or even both receptors, the organisms were still capable of maintaining proper organ function and preserving physiological homeostasis under basal conditions. This highlights the inherent plasticity of regulatory systems, which may compensate for the absence of specific receptors through alternative mechanisms. However, such compensatory responses appear to be insufficient when challenged by a pathological stimulus. In such cases, the defensive responses are disrupted, leading to pathological outcomes that fully receptor-competent organisms can avoid. These insights may be extrapolated to other physiological systems, suggesting that certain receptor- or peptide-dependent deficits may only manifest under stress or pathological conditions, remaining undetectable under normal circumstances.

### 4.5. Limitations

The present study has several limitations. The mechanistic interpretation is based on morphological, molecular, and localization data rather than direct analysis of intracellular signaling pathways. In addition, immunofluorescence-based localization should be interpreted with caution due to the possibility of non-specific staining and the relatively weak and sparse TRPA1 immunoreactivity, particularly in neuronal structures. Therefore, these localization findings should be considered primarily descriptive rather than definitive. However, the use of appropriate control procedures and repeated observations supported the distinction between specific signals and background fluorescence. Furthermore, the study did not include direct physiological measurements of gastric function, such as acid secretion or motility.

Although a graded pattern of response was observed across the studied groups, not all differences between knockout models reached statistical significance. Therefore, the interpretation of potential interactions between TRPA1 and TRPV1 should be approached with caution.

Taken together, the combination of genetic knockout models, analysis of inflammatory responses, and detailed anatomical localization within both neuronal and non-neuronal structures provides complementary evidence suggesting the involvement of TRPA1 and TRPV1 in gastric mucosal protection. Future studies should address receptor-specific signaling pathways and physiological responses in greater detail.

## 5. Conclusions

The present study demonstrates that both TRPA1 and TRPV1 receptors contribute to gastric mucosal protection against ethanol-induced injury. Importantly, the protective effects of these receptors differ in magnitude: TRPV1 exerts a modest protective effect, whereas TRPA1 provides stronger protection, and the combined deficiency results in the most severe gastric damage.

The observed time-dependent changes in receptor expression suggest adaptive responses of gastric tissue to ethanol exposure. In addition, the localization of TRPA1 and TRPV1 in both neuronal and non-neuronal gastric cell populations indicates that receptor-mediated protection may involve multiple cellular mechanisms within the gastric wall. Importantly, no pathological changes were observed in receptor-deficient animals under physiological conditions, indicating that these receptors are not essential for basal gastric homeostasis but become critical under ethanol-induced stress.

These findings highlight the importance of TRP receptors in maintaining gastric mucosal integrity and provide a basis for further studies on their role in gastrointestinal defense mechanisms. Moreover, the observed differences between ethanol- and indomethacin-induced gastric injury indicate that distinct pathological stimuli may engage different TRP-dependent mechanisms, underscoring the need for condition-specific investigations.

## Figures and Tables

**Table 1:** Primer sequences used for qPCR analysis.

Studied Gene	Sequence of Origin	Primer Sequence (5′->3′)	Product Length (bp)
*Gapdh*	NM_008084.4	Forward: AGCCTCGTCCCGTAGACAAAAT Reverse: CCGTGAGTGGAGTCATACTGGA	175
*Trpv1*	NM_001001445.2	Forward: GTGGACAGCTACAGTGAGATAC Reverse: GCCACATACTCCTTGCGAT	95
*Trpa1*	NM_177781.5	Forward: ATCCAAATAGACCCAGGCACG Reverse: AAGCATGTGTCAATGTTTGGTACT	101
*IL6*	NM_031168	Forward: TACCACTTCACAAGTCGGAGGC Reverse: CTGCAAGTGCATCATCGTTGTTC	116
*IL1* *β*	NM_008361.4	Forward: TTCAGGCAGGCAGTATCACTC Reverse: GAAGGTCCACGGGAAAGACAC	75

## Data Availability

The data presented in this study are available from the corresponding author upon reasonable request.

## Abbreviations

The following abbreviations are used in this manuscript:

dKO	double knockout animals
IF	immunofluorescent
KO	knockout animal
Q-PCR	real-time PCR
TRP	transient receptor potential cation channel
TRPA1	transient receptor potential ankyrin 1
TRPV1	transient receptor potential vanilloid 1
WT	wild-type mice

## References

[1] Matsuda H., Li Y., Yoshikawa M. (1999). Roles of capsaicin-sensitive sensory nerves, endogenous nitric oxide, sulfhydryls, and prostaglandins in gastroprotection by momordin Ic, an oleanolic acid oligoglycoside, on ethanol-induced gastric mucosal lesions in rats. Life Sci..

[2] Matsuda H., Li Y., Yoshikawa M. (1999). Gastroprotections of escins Ia, Ib, IIa, and IIb on ethanol-induced gastric mucosal lesions in rats. Eur. J. Pharmacol..

[3] Matsuda H., Ochi M., Nagatomo A., Yoshikawa M. (2007). Effects of allyl isothiocyanate from horseradish on several experimental gastric lesions in rats. Eur. J. Pharmacol..

[4] Morikawa T., Matsuda H., Yamaguchi I., Pongpiriyadacha Y., Yoshikawa M. (2004). New Amides and gastroprotective constituents from the fruit of *Piper chaba*. Planta Med..

[5] Pongpiriyadacha Y., Matsuda H., Morikawa T., Asao Y., Yoshikawa M. (2003). Protective effects of polygodial on gastric mucosal lesions induced by necrotizing agents in rats and the possible mechanisms of action. Biol. Pharm. Bull..

[6] Shin I.S., Masuda H., Naohide K. (2004). Bactericidal activity of wasabi (*Wasabia japonica*) against *Helicobacter pylori*. Int. J. Food Microbiol..

[7] Bai Y.F., Xu H. (2000). Protective action of piperine against experimental gastric ulcer. Acta Pharmacol. Sin..

[8] Mózsik G., Szolcsányi J., Rácz I. (2005). Gastroprotection induced by capsaicin in healthy human subjects. World J. Gastroenterol..

[9] Smani T., Shapovalov G., Skryma R., Prevarskaya N., Rosado J.A. (2015). Functional and physiopathological implications of TRP channels. Biochim. Biophys. Acta.

[10] Yarushkina N.I., Sudalina M.N., Punin Y.M., Filaretova L.P. (2018). Vulnerability of gastric and small intestinal mucosa to ulcerogenic action of indomethacin in C57/BL6/J mice and transient receptor potential channel vanilloid type 1 knockout mice. J. Physiol. Pharmacol..

[11] Xiang Y., Xu X., Zhang T., Wu X., Fan D., Hu Y., Ding J., Yang X., Lou J., Du Q. (2022). Beneficial effects of dietary capsaicin in gastrointestinal health and disease. Exp. Cell Res..

[12] Zhang W., Zhang Y., Fan J., Feng Z., Song X. (2024). Pharmacological activity of capsaicin: Mechanisms and controversies (Review). Mol. Med. Rep..

[13] Bertin S., Aoki-Nonaka Y., Lee J., De Jong P.R., Kim P., Han T., Yu T., To K., Takahashi N., Boland B.S. (2017). The TRPA1 ion channel is expressed in CD4+ t cells and restrains T-cell-mediated colitis through inhibition of TRPV1. Gut.

[14] Cseko K., Beckers B., Keszthelyi D., Helyes Z. (2019). Role of TRPV1 and TRPA1 Ion Channels in Inflammatory Bowel Diseases: Potential Therapeutic Targets?. Pharmaceuticals.

[15] Utsumi D., Matsumoto K., Tsukahara T., Amagase K., Tominaga M., Kato S. (2018). Transient receptor potential vanilloid 1 and transient receptor potential ankyrin 1 contribute to the progression of colonic inflammation in dextran sulfate sodium-induced colitis in mice: Links to calcitonin gene-related peptide and substance P. J. Pharmacol. Sci..

[16] Massa F., Sibaev A., Marsicano G., Blaudzun H., Storr M., Lutz B. (2006). Vanilloid receptor (TRPV1)-deficient mice show increased susceptibility to dinitrobenzene sulfonic acid induced colitis. J. Mol. Med..

[17] Kun J., Szitter I., Kemény Á., Perkecz A., Kereskai L., Pohóczky K., Vincze Á., Gódi S., Szabó I., Szolcsányi J. (2014). Upregulation of the transient receptor potential ankyrin 1 ion channel in the inflamed human and mouse colon and its protective roles. PLoS ONE.

[18] Meng J., Wang J., Steinhoff M., Dolly J.O. (2016). TNFalpha induces co-trafficking of TRPV1/TRPA1 in VAMP1-containing vesicles to the plasmalemma via Munc18-1/syntaxin1/SNAP-25 mediated fusion. Sci. Rep..

[19] Kobayashi K., Fukuoka T., Obata K., Yamanaka H., Dai Y., Tokunaga A., Noguchi K. (2005). Distinct expression of TRPM8, TRPA1, and TRPV1 mRNAs in rat primary afferent neurons with adelta/c-fibers and colocalization with trk receptors. J. Comp. Neurol..

[20] Story G.M., Peier A.M., Reeve A.J., Eid S.R., Mosbacher J., Hricik T.R., Earley T.J., Hergarden A.C., Andersson D.A., Hwang S.W. (2003). ANKTM1, a TRP-like channel expressed in nociceptive neurons, is activated by cold temperatures. Cell.

[21] Numazaki M., Tominaga M. (2004). Nociception and TRP Channels. Curr. Drug Targets CNS Neurol. Disord..

[22] Mickle A.D., Shepherd A.J., Mohapatra D.P. (2015). Sensory TRP channels: The key transducers of nociception and pain. Prog. Mol. Biol. Transl. Sci..

[23] Hung C.Y., Tan C.H. (2018). TRP Channels in Nociception and Pathological Pain. Adv. Exp. Med. Biol..

[24] Holzer P., Sametz W. (1986). Gastric mucosal protection against ulcerogenic factors in the rat mediated by capsaicin-sensitive afferent neurons. Gastroenterology.

[25] Holzer P. (1998). Neural emergency system in the stomach. Gastroenterology.

[26] Cho H.J., Callaghan B., Bron R., Bravo D.M., Furness J.B. (2014). Identification of enteroendocrine cells that express TRPA1 channels in the mouse intestine. Cell Tissue Res..

[27] Barton J.R., Londregan A.K., Alexander T.D., Entezari A.A., Covarrubias M., Waldman S.A. (2023). Enteroendocrine cell regulation of the gut-brain axis. Front. Neurosci..

[28] Kemény Á., Kodji X., Horváth S., Komlódi R., Szőke É., Sándor Z., Perkecz A., Gyömörei C., Sétáló G., Kelemen B. (2018). TRPA1 Acts in a Protective Manner in Imiquimod-Induced Psoriasiform Dermatitis in Mice. J. Investig. Dermatol..

[29] Tékus V., Horváth Á., Hajna Z., Borbély É., Bölcskei K., Boros M., Pintér E., Helyes Z., Petho G., Szolcsányi J. (2016). Noxious heat threshold temperature and pronociceptive effects of allyl isothiocyanate (mustard oil) in TRPV1 or TRPA1 gene-deleted mice. Life Sci..

[30] Andrade M.C., Menezes J.S., Cassali G.D., Martins-Filho O.A., Cara D.C., Faria A.M. (2006). Alcohol-induced gastritis prevents oral tolerance induction in mice. Clin. Exp. Immunol..

[31] Zalecki M. (2012). Localization and neurochemical characteristics of the extrinsic sympathetic neurons projecting to the pylorus in the domestic pig. J. Chem. Neuroanat..

[32] Franke-Radowiecka A., Bossowska A. (2025). Histomorphological changes in lumbar sympathetic chain ganglia of the female pig during prenatal development. Pol. J. Vet. Sci..

[33] Franke-Radowiecka A., Demus N.V., Bossowska A. (2024). Innervation of the female internal genital organs in 12-week-old porcine foetuses. Pol. J. Vet. Sci..

[34] Dudek A., Sienkiewicz W., Lepiarczyk E., Kaleczyc J. (2024). Immunohistochemical properties of motoneurons supplying the porcine trapezius muscle. Pol. J. Vet. Sci..

[35] Holzer P., Lippe I.T. (1988). Stimulation of afferent nerve endings by intragastric capsaicin protects against ethanol-induced damage of gastric mucosa. Neuroscience.

[36] Csekő K., Pécsi D., Kajtár B., Hegedűs I., Bollenbach A., Tsikas D., Szabó I.L., Szabó S., Helyes Z. (2020). Upregulation of the trpa1 ion channel in the gastric mucosa after iodoacetamide-induced gastritis in rats: A potential new therapeutic target. Int. J. Mol. Sci..

[37] Ren H., Meng Q., Yepuri N., Du X., Sarpong J.O., Cooney R.N. (2018). Protective effects of glutathione on oxidative injury induced by hydrogen peroxide in intestinal epithelial cells. J. Surg. Res..

[38] Slomiany A., Piotrowski E., Piotrowski J., Slomiany B.L. (2000). Impact of ethanol on innate protection of gastric mucosal epithelial surfaces and the risk of injury. J. Physiol. Pharmacol..

[39] Jain P., Materazzi S., De Logu F., Degl’Innocenti D.R., Fusi C., Li Puma S., Marone I.M., Coppi E., Holzer P., Geppetti P. (2020). Transient receptor potential ankyrin 1 contributes to somatic pain hypersensitivity in experimental colitis. Sci. Rep..

[40] Szolcsanyi J., Bartho L. (2001). Capsaicin-sensitive afferents and their role in gastroprotection: An update. J. Physiol..

[41] Larauche M., Anton P.M., Peiro G., Eutamene H., Bueno L., Fioramonti J. (2004). Role of capsaicin-sensitive afferent nerves in different models of gastric inflammation in rats. Auton. Neurosci..

[42] Zhu M.X. (2025). TRP-Mediated Signaling.

[43] Pascual-Mato M., Garate Vinas G., Munoz San Martin M., Gonzalez-Quintanilla V., Crespo J., Rivero Tirado M., Pascual Gomez J. (2025). Calcitonin gene-related peptide (CGRP) in the pathophysiology of gastrointestinal disorders—A key mediator in the gut-brain axis. Rev. Esp. Enferm. Dig..

[44] Khalil M., Alliger K., Weidinger C., Yerinde C., Wirtz S., Becker C., Engel M.A. (2018). Functional Role of Transient Receptor Potential Channels in Immune Cells and Epithelia. Front. Immunol..

[45] Yan Q., Gao C., Li M., Lan R., Wei S., Fan R., Cheng W. (2024). TRP Ion Channels in Immune Cells and Their Implications for Inflammation. Int. J. Mol. Sci..

[46] Fukushima K., Aoi Y., Kato S., Takeuchi K. (2006). Gastro-protective action of lafutidine mediated by capsaicin-sensitive afferent neurons without interaction with TRPV1 and involvement of endogenous prostaglandins. World J. Gastroenterol..

[47] Lee I.O., Lee K.H., Pyo J.H., Kim J.H., Choi Y.J., Lee Y.C. (2007). Anti-inflammatory effect of capsaicin in Helicobacter pylori-infected gastric epithelial cells. Helicobacter.

[48] Zhao X., Dong B., Friesen M., Liu S., Zhu C., Yang C. (2021). Capsaicin Attenuates Lipopolysaccharide-Induced Inflammation and Barrier Dysfunction in Intestinal Porcine Epithelial Cell Line-J2. Front. Physiol..

[49] Faussone-Pellegrini M.S., Taddei A., Bizzoco E., Lazzeri M., Vannucchi M.G., Bechi P. (2005). Distribution of the vanilloid (capsaicin) receptor type 1 in the human stomach. Histochem. Cell Biol..

[50] Holzer P. (2011). TRP channels in the digestive system. Curr. Pharm. Biotechnol..

[51] Holzer P. (2011). Transient receptor potential (TRP) channels as drug targets for diseases of the digestive system. Pharmacol. Ther..

[52] Van Liefferinge E., Van Noten N., Degroote J., Vrolix G., Van Poucke M., Peelman L., Van Ginneken C., Roura E., Michiels J. (2020). Expression of transient receptor potential ankyrin 1 and transient receptor potential vanilloid 1 in the gut of the peri-weaning pig is strongly dependent on age and intestinal site. Animals.

[53] Zalecki M., Podlasz P., Pidsudko Z., Wojtkiewicz J., Kaleczyc J. (2012). Vagal projections to the pylorus in the domestic pig (*Sus scrofa domestica*). Auton. Neurosci..

[54] Zalecki M. (2014). Extrinsic primary afferent neurons projecting to the pylorus in the domestic pig—Localization and neurochemical characteristics. J. Mol. Neurosci..

[55] Schicho R., Florian W., Liebmann I., Holzer P., Lippe I.T. (2004). Increased expression of TRPV1 receptor in dorsal root ganglia by acid insult of the rat gastric mucosa. Eur. J. Neurosci..

[56] Bertin S., Aoki-Nonaka Y., De Jong P.R., Nohara L.L., Xu H., Stanwood S.R., Srikanth S., Lee J., To K., Abramson L. (2014). The ion channel TRPV1 regulates the activation and proinflammatory properties of CD4^+^ T cells. Nat. Immunol..

[57] Fernandes E.S., Fernandes M.A., Keeble J.E. (2012). The functions of TRPA1 and TRPV1: Moving away from sensory nerves. Br. J. Pharmacol..

[58] Luu D.D., Owens A.M., Mebrat M.D., Van Horn W.D. (2023). A molecular perspective on identifying TRPV1 thermosensitive regions and disentangling polymodal activation. Temperature.

[59] Dhaka A., Uzzell V., Dubin A.E., Mathur J., Petrus M., Bandell M., Patapoutian A. (2009). TRPV1 is activated by both acidic and basic pH. J. Neurosci..

[60] Takaishi M., Uchida K., Suzuki Y., Matsui H., Shimada T., Fujita F., Tominaga M. (2016). Reciprocal effects of capsaicin and menthol on thermosensation through regulated activities of TRPV1 and TRPM8. J. Physiol. Sci..

[61] Blednov Y.A., Harris R.A. (2009). Deletion of vanilloid receptor (TRPV1) in mice alters behavioral effects of ethanol. Neuropharmacology.

[62] May D., Baastrup J., Nientit M.R., Binder A., Schunke M., Baron R., Cascorbi I. (2012). Differential expression and functionality of TRPA1 protein genetic variants in conditions of thermal stimulation. J. Biol. Chem..

[63] Bautista D.M., Jordt S.E., Nikai T., Tsuruda P.R., Read A.J., Poblete J., Yamoah E.N., Basbaum A.I., Julius D. (2006). TRPA1 mediates the inflammatory actions of environmental irritants and proalgesic agents. Cell.

[64] Nilius B., Appendino G., Owsianik G. (2012). The transient receptor potential channel TRPA1: From gene to pathophysiology. Pflug. Arch..

[65] Bautista D.M., Pellegrino M., Tsunozaki M. (2013). TRPA1: A gatekeeper for inflammation. Annu. Rev. Physiol..

[66] Engel M.A., Leffler A., Niedermirtl F., Babes A., Zimmermann K., Filipovic M.R., Izydorczyk I., Eberhardt M., Kichko T.I., Mueller-Tribbensee S.M. (2011). TRPA1 and substance P mediate colitis in mice. Gastroenterology.

[67] Kimball E.S., Prouty S.P., Pavlick K.P., Wallace N.H., Schneider C.R., Hornby P.J. (2007). Stimulation of neuronal receptors, neuropeptides and cytokines during experimental oil of mustard colitis. Neurogastroenterol. Motil..

[68] Pagano E., Romano B., Iannotti F.A., Parisi O.A., D’Armiento M., Pignatiello S., Coretti L., Lucafo M., Venneri T., Stocco G. (2019). The non-euphoric phytocannabinoid cannabidivarin counteracts intestinal inflammation in mice and cytokine expression in biopsies from UC pediatric patients. Pharmacol. Res..

[69] Nozawa K., Kawabata-Shoda E., Doihara H., Kojima R., Okada H., Mochizuki S., Sano Y., Inamura K., Matsushime H., Koizumi T. (2009). TRPA1 regulates gastrointestinal motility through serotonin release from enterochromaffin cells. Proc. Natl. Acad. Sci. USA.

[70] Poole D.P., Pelayo J.C., Cattaruzza F., Kuo Y., Gai G., Chiu J.V., Bron R., Furness J.B., Grady E.F., Bunnett N.W. (2011). Transient receptor potential ankyrin 1 is expressed by inhibitory motoneurons of the mouse intestine. Gastroenterology.

[71] Story G.M., Gereau R.W.t. (2006). Numbing the senses: Role of TRPA1 in mechanical and cold sensation. Neuron.

[72] Heitzmann D., Warth R. (2007). No potassium, no acid: K+ channels and gastric acid secretion. Physiology.

[73] Staruschenko A., Jeske N.A., Akopian A.N. (2010). Contribution of TRPV1-TRPA1 interaction to the single channel properties of the TRPA1 channel. J. Biol. Chem..

